# Dynamic Combinatorial Chemistry of Ditellurides

**DOI:** 10.1002/chem.202501291

**Published:** 2025-05-28

**Authors:** Christian D. Fisker, Jordi Poater, F. Matthias Bickelhaupt, Jasmin Mecinović

**Affiliations:** ^1^ Department of Physics, Chemistry and Pharmacy University of Southern Denmark Campusvej 55 5230 Odense Denmark; ^2^ ICREA, Passeig Lluís Companys 23 08010 Barcelona Spain; ^3^ Departament de Química Inorgànica i Orgànica & IQTCUB Universitat de Barcelona Martí i Franquès 1–11 08028 Barcelona Spain; ^4^ Department of Chemistry and Pharmaceutical Sciences, Amsterdam Institute of Molecular and Life Sciences (AIMMS) Vrije Universiteit Amsterdam De Boelelaan 1108 1081 HZ Amsterdam The Netherlands; ^5^ Institute for Molecules and Materials Radboud University Heyendaalseweg 135 6525 AJ Nijmegen The Netherlands; ^6^ Department of Chemical Sciences University of Johannesburg Auckland Park 2006 Johannesburg South Africa

**Keywords:** ^125^Te NMR spectroscopy, chalcogen‐chalcogen exchange, DFT calculations, ditelluride, dynamic combinatorial chemistry

## Abstract

Dynamic combinatorial chemistry enables the exploration of reversible chemical exchange reactions. Here, we report studies on the dynamic Te─Te bond exchange of chemically and structurally diverse ditellurides using ^125^Te NMR spectroscopy. The results revealed rapid and unbiased equilibration, forming a consistent 1:1 distribution of reactants and products across all chemical systems. Extending to multi‐component mixtures, we observed robust equilibrium formation even in complex systems involving multiple ditelluride species that can be easily distinguished by ^125^Te NMR spectroscopy. In line with the NMR analyses showing that the Te─Te bond exchange efficiently proceeds in various solvents, at very low temperatures, in the absence of visible light, and in the presence of radical scavengers, quantum chemical analyses revealed that the ditelluride‐ditelluride exchange likely proceeds via a non‐synchronous concerted mechanism and not via an alternative radical mechanism. Integrated NMR spectroscopic and quantum chemical analyses also showed that the efficiency of the chalcogen‐chalcogen exchange involving ditellurides follows the trend Te‐Te > Te‐Se > Te‐S. Overall, the results indicate that ditellurides are excellent dynamic systems that may find applications across molecular sciences.

## Graphical abstract

Ditelluride exchange. Chemically and structurally diverse ditellurides undergo a rapid Te‐Te exchange under mild reaction conditions in the absence of an external stimulus, indicating that ditellurides could find application in various dynamic molecular systems.

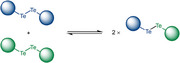

## 1 Introduction

Dynamic combinatorial chemistry (DCC) is a powerful chemical method that uses reversible chemical reactions to create dynamic combinatorial libraries (DCLs).^[^ ^1^ ^]^ These libraries are made by combining different molecular building blocks under conditions that allow covalent bonds to form and break reversibly.^[^ ^1^ ^]^ The continuous bond exchange process allows the system to adjust itself and reach a balanced state, or equilibrium. DCC has been found useful in many areas of chemistry, including drug discovery, molecular recognition, and materials science.^[^ ^2^ ^]^ In drug discovery, DCC has been used to help create libraries of molecules that can adapt and bind strongly to specific targets, such as enzymes or receptors.^[^ ^2c,e^ ^]^ The reversible nature of these reactions allows potential drug molecules to optimize their configurations, enabling the development of novel inhibitors and binding ligands for challenging therapeutic targets creating target‐specific potent inhibitors. In molecular recognition, DCC enables the selection of highly specific binding partners for designing sensors, molecular assemblies, and other precision tools.^[^ ^2a,d^ ^]^ In materials science, DCC supports the development of adaptive materials that can respond to environmental changes, such as variations in temperature, pH, or light, by reorganizing their structures dynamically.^[^ ^2b^ ^]^ Additionally, DCC also enables the creation of catalysts that can perform several different chemical reactions, often adapting to changes in their surroundings.^[^ ^3^ ^]^

**Figure 1:**
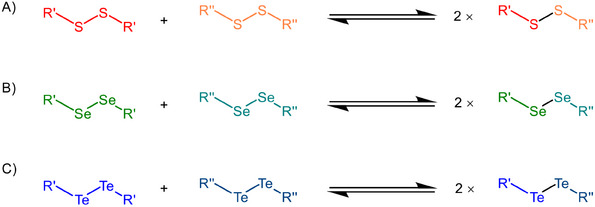
Dynamic combinatorial chemistry of A) disulfides, B) diselenides, and C) ditellurides.

A range of reversible dynamic reactions has been researched each with unique strengths and applications. Imine formation reaction, for example, uses amines and aldehydes, or less commonly ketones.^[^ ^4^ ^]^ This reaction is widely used due to its reactivity under mild conditions in aqueous solution. However, the imines are inherently labile and unstable, and thus require reducing agents before direct analysis can be undergone. Related hydrazones, formed between hydrazides and aldehydes under acidic conditions, are more stable for analysis.^[^ ^5^ ^]^ Due to their slow formation at neutral pH, hydrazones are often not compatible with biological systems. Boronate ester‐based dynamic systems, on the other hand, have been particularly useful in aqueous systems, enabling the identification of protein inhibitors.^[^ ^6^ ^]^ Another well‐established DCC system is the thiol/disulfide (S─S) bond exchange, which is relevant due to its ability to reach equilibrium in aqueous environment (Figure 1A).^[^ ^7^ ^]^ Thiol/disulfide exchange occurs when nucleophilic thiolates attack pre‐existing electrophilic disulfide bonds, facilitating continuous bond rearrangement. Similarly, diselenide (Se─Se) bonds—another chalcogen‐based system—can undergo exchange in a similar manner but offer certain advantages over disulfide bonds (Figure 1B).^[^ ^8^ ^]^ Diselenide bonds have a lower bond dissociation energy than disulfide bonds, which results in a more reactive DCC system that allows for a more dynamic library that equilibrates more rapidly. Additionally, the diselenide exchange occurs in aqueous solution at neutral to slightly acidic pH levels, given that the pK_a_ of selenols is around 5.2 compared to thiols at 8.2, making diselenide systems more suitable for biological applications. Transitioning to the heavier chalcogen tellurium introduces another class of highly reactive building blocks for DCLs. Tellurium, the heaviest stable chalcogen, forms Te─Te bonds with a bond dissociation energy of 126 kJ mol^−1^, significantly lower than that of Se‐Se bonds (172 kJ mol^−1^) and S─S bonds (240 kJ mol^−1^).^[^ ^9^ ^]^ This lower bond dissociation energy makes ditellurides exceptionally suitable for dynamic exchange reactions, as they can break and reform bonds more readily (Figure 1C). For example, ditelluride‐containing polyurethane has been synthesized and shown to exhibit self‐healing properties at room temperature without external intervention.^[^ ^10^ ^]^ This remarkable self‐repair capability is due to the reversible exchange of ditelluride bonds, which allows the material to spontaneously repair itself. In this work, we explore the dynamic nature of diaryl‐ and dialkyl ditellurides to better understand their reactivity in dynamic combinatorial chemistry. Using ^125^Te NMR spectroscopy and quantum chemical analyses, we show that these systems quickly form equilibrium and exchange bonds consistently in the absence of an external stimulus. These results highlight the potential of ditelluride‐based systems as adaptable building blocks for dynamic combinatorial chemistry across chemical sciences.

## 2 Results and Discussion

A panel of ditelluride compounds was synthesized using either bromo‐ or iodobenzene moieties. Lithium‐halogen exchange was performed using t‐BuLi, whereas metallic tellurium was subsequently added to form the diaryl ditellurides (Figure S1). For the preparation of dialkyl ditellurides, alkyl organolithium compounds were used, such that t‐BuLi was not required, and that the substitution to the ditelluride moiety occurred in a single step (Figure S2). The synthesized ditellurides were used for the creation of the dynamic combinatorial libraries, to determine their ability to efficiently form an equilibrium. We envisioned that ^125^Te NMR spectroscopy might serve as an excellent method for monitoring the equilibrium between ditellurides. Initially, we examined commercial and synthetic ditellurides as standards in CDCl_3_ by ^125^Te NMR, demonstrating that homo‐ditellurides display different ^125^Te chemical shifts and could be distinguished when present together in mixtures (Table S1). A molar equivalent of the ditelluride compounds was mixed to determine the rate and extent of the equilibrium formation. *Para*‐substitution was specifically chosen for the aromatic ditellurides to minimize steric effects on the tellurium centers, while still allowing for exploration of the electronic effects.

**Figure 2:**
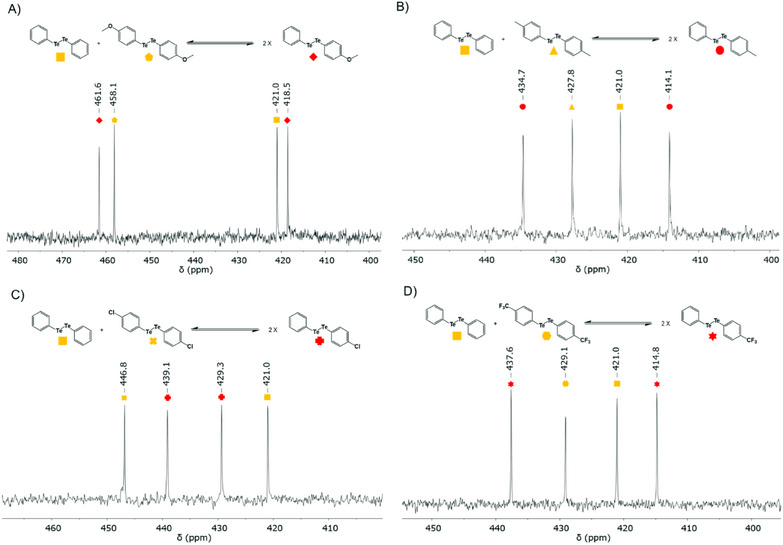
Dynamic combinatorial chemistry of ditellurides. ^125^Te NMR spectra showing the formation of hetero‐ditellurides from the corresponding homo‐ditellurides within 20 min at room temperature in CDCl_3_. A) Ph_2_Te_2_ and (*p‐*MeOPh)_2_Te_2_, B) Ph_2_Te_2_ and (*p‐*MePh)_2_Te_2_, C) Ph_2_Te_2_ and (*p‐*ClPh)_2_Te_2_, D) Ph_2_Te_2_ and (*p‐*CF_3_Ph)_2_Te_2_.

Four different *para*‐substituted diaryl ditellurides were initially used, with substitutions spanning a range of electron‐donating and electron‐withdrawing groups to assess potential differences in reactivity and equilibrium behavior. The results of four experiments illustrate the rapid formation of an equilibrium distribution of hetero‐ditellurides, exhibiting two new tellurium signals in ^125^Te NMR spectra within 20 min and overnight at room temperature in CDCl_3_ (Figure 2; Figures S3–S5). As expected, the resulting equilibrium exists in a 1:1 distribution between the reacting homo‐ditellurides and the hetero‐ditelluride products. No significant differences in reactivity were observed amongst the four independent systems, nor was there any clear pattern in the chemical shift for formation of new peaks, although the newly formed peaks of hetero‐ditelluride closest to homo‐ditelluride peaks correspond to the tellurium species they originate from. For example, Ph_2_Te_2_ was standardized at 421.0 ppm for each of the experiments. Mixing Ph_2_Te_2_ (421.0 ppm) and (*p‐*MeOPh)_2_Te_2_ (458.1 ppm) resulted in the formation of PhTeTe(*p‐*MeOPh) possessing two new Te peaks (461.6 and 418.5 ppm, Figure 2A). Similarly, when Ph_2_Te_2_ (421.0 ppm) and (*p‐*MePh)_2_Te_2_ (427.8 ppm) were mixed, a newly formed PhTeTe(*p‐*MePh) showed two new Te signals (434.7 and 414.1 ppm, Figure 2B). Moreover, the reaction between Ph_2_Te_2_ and (*p‐*ClPh)_2_Te_2_ produced PhTeTe(*p‐*ClPh) (439.1 and 429.3 ppm, Figure 2C), whereas Ph_2_Te_2_ and (*p‐*CF_3_Ph)_2_Te_2_ reacted to produce PhTeTe(*p‐*CF_3_Ph) (437.6 and 414.8 ppm, Figure 2D) within 20 min at room temperature.

Subsequently, the additional pairs of *para*‐substituted diaryl ditellurides were mixed separately, one pair at a time, to further assess their equilibrium behavior. Each combination formed a 1:1 equilibrium within 20 min and remained stable overnight at room temperature, with two new hetero‐ditelluride signals observed in the ^125^Te NMR spectra (Figures S3–S5). The new peaks appeared near the homo‐ditelluride signals of their respective tellurium species, and no significant differences in reactivity or chemical shift patterns were observed. The only system where the newly established hetero‐ditelluride exhibits peaks “inside” the homo‐ditelluride peaks is the *para*‐chloro substituted diaryl ditelluride and another system consisting of the *para*‐chloro substituted diaryl ditelluride and the *para*‐methyl substituted diaryl ditelluride (Figures 2; Figures S3–S5).

**Figure 3:**
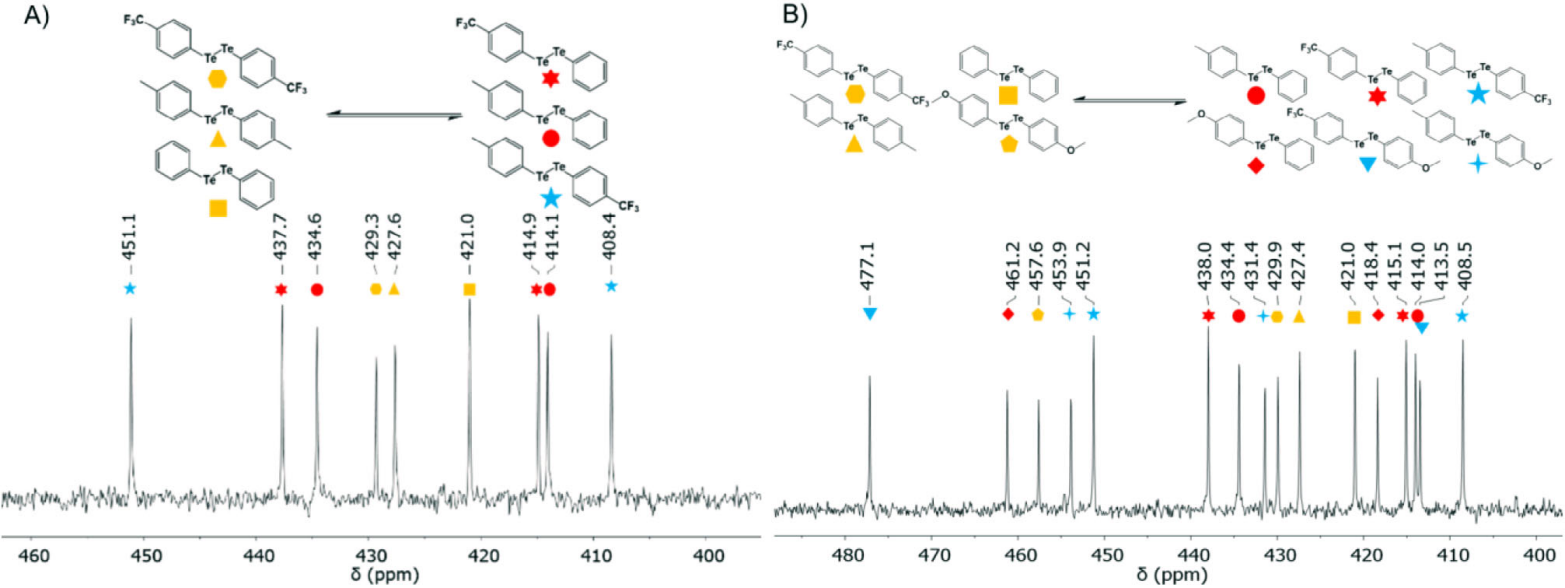
^125^Te NMR spectra showing the dynamic formation of new three‐component and four‐component equilibrium states. A) Molar equal amount mixture of three diaryl ditellurides consisting of (*p‐*CF_3_Ph)_2_Te_2_, (*p‐*MePh)_2_Te_2_, and Ph_2_Te_2_ added in that order. B) Molar equal amount mixture of four diaryl ditellurides consisting of (*p‐*CF_3_Ph)_2_Te_2_, (*p‐*MePh)_2_Te_2_, Ph_2_Te_2_, and (*p‐*MeOPh)_2_Te_2_ added in that order.

By introducing an additional ditelluride species to a preestablished two‐system of *para*‐methyl diaryl ditelluride and *para*‐trifluoromethyl diaryl ditelluride, a new equilibrium state between three homo‐ditellurides can be formed. In this specific experiment, the equilibrium was intentionally disrupted by the addition of a molar equivalent amount of diphenyl ditelluride. The results demonstrated that a new three‐way equilibrium showing six distinct signals among the three ditelluride species formed almost instantaneously at room temperature in CDCl_3_ (Figures 3A; Figure S6). This rapid equilibration indicates that the dynamic exchange reactions between ditelluride species are unbiased, with no preference toward any particular direction in the equilibrium state. The addition of another homo‐ditelluride further pressures the limit of the formation of equilibrium. When *para‐*methoxy diaryl ditelluride was introduced in equimolar amounts to the existing mixture, it disturbed the balance even further and led to the formation of a new, four‐way equilibrium state (Figure 3B). The final equilibrium composition was observed to be unaffected by the order of addition. Whether *para‐*methyl ditelluride is introduced first, followed by *para‐*trifluoromethyl ditelluride, diphenyl ditelluride, and *para‐*methoxy ditelluride, or if the sequence is reversed, the system always reaches the same equilibrium state (Figures S7–S10). The resulting ^125^Te NMR spectrum shows 16 distinct peaks ranging from 408 to 477 ppm, including four from the homo‐ditellurides and 12 from the hetero‐ditellurides (Figure 3B). No significant differences were found between the two spectra portraying the two systems of four species added in reverse order, and also when all four homo‐ditellurides were mixed together at the same time (Figures S7–S10).

**Figure 4:**
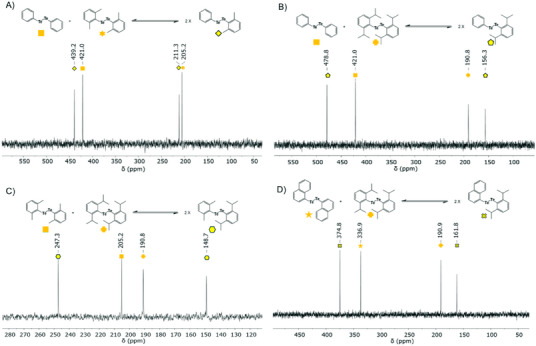
^125^Te NMR spectra showing the formation of the sterically hindered ditelluride species within 20 min at room temperature in CDCl_3_. A) A molar equivalent mixture of Ph_2_Te2 and (*o,o*‐Me_2_Ph)2Te2. B) A molar equivalent mixture of Ph_2_Te_2_ and (*o,o*‐iPr_2_Ph)_2_Te_2_. C) A molar equivalent mixture of (*o,o*‐Me_2_Ph)_2_Te_2_ and (*o,o*‐iPr_2_Ph)_2_Te_2_. D) A molar equivalent mixture of (*o,o*‐iPr_2_Ph)_2_Te_2_ and Naph_2_Te_2_.

To investigate strategies to prevent or slow down the exchange of ditellurides, two sterically demanding *ortho*‐disubstituted diaryl ditelluride compounds and one bulky naphthalene‐based ditelluride were synthesized. The *ortho* substituted compounds chosen to be synthesized were di(*o,o*‐dimethylphenyl) ditelluride and di(*o,o*‐diisopropylphenyl) ditelluride. Despite the increased steric bulk, the results showed no difference in the reaction rate and no shift in the equilibrium of the ditelluride formation (Figure 4). The results show that the Te─Te exchange mechanism is either not significantly affected by steric hindrance or that the steric effect of the substituents was not enough to hinder the exchange. The positions of the newly formed hetero‐ditelluride peaks differ from those in conventional systems, showing a higher chemical shift rather than appearing between the homo‐ditelluride peaks. Notably, newly formed ^125^Te signals were observed within 20 min at room temperature for highly sterically demanding (*o,o*‐Me_2_Ph)TeTe(*o,o*‐iPr_2_Ph) (247.3 and 148.7 ppm, Figure 4C) and NaphTeTe(*o,o*‐iPr_2_Ph) (374.8 and 161.8 ppm, Figure 4D).

**Figure 5:**
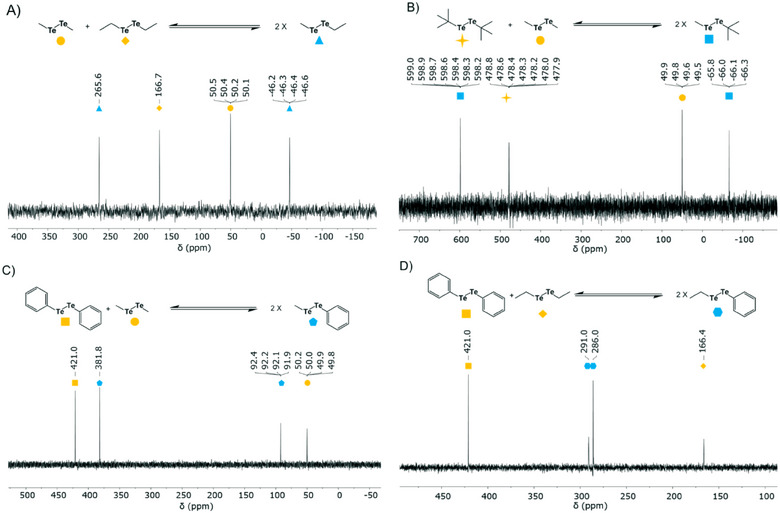
^125^Te NMR spectra of ditelluride systems containing both aromatic and aliphatic ditellurides, within 20 min at room temperature in CDCl_3_. A) A molar equivalent mixture of Me_2_Te_2_ and Et_2_Te_2_. B) A molar equivalent mixture of Me_2_Te_2_ and tBu_2_Te_2_ C) A molar equivalent mixture of Ph_2_Te_2_ and Me_2_Te_2_. D) A molar equivalent mixture of Ph_2_Te_2_ and Et_2_Te_2_.

To investigate whether aliphatic ditellurides exhibit similar behavior, dimethyl ditelluride and diethyl ditelluride, the two simplest dialkyl ditellurides, were mixed in equimolar amounts. The results demonstrated that hetero‐aliphatic ditellurides also formed an instant equilibrium at room temperature (Figure 5A,B), like the ones observed with their aromatic counterparts. This observation shows that the dynamic exchange properties of ditellurides are independent of the nature of the side chain and that both types can achieve rapid equilibrium under the same conditions. To further explore the behavior of dialiphatic and diaromatic ditellurides, experiments were conducted by mixing dialkyl ditellurides with diaryl ditellurides in equimolar amounts. Dimethyl ditelluride and diethyl ditelluride were each combined with diphenyl ditelluride, and the mixtures were monitored by ^125^Te NMR spectroscopy (Figure 5C,D). The unequal distribution of the peak size of tellurium of the diphenyl species in comparison to the aliphatic counterparts is caused by the Te‐H coupling of dimethyl ditelluride, resulting in a quadruplet split of signal and reduced apparent signal intensity with the same overall integration, however, the intensity of chemically similar Te species indicates that the equilibrium is reached rapidly.

Having observed an efficient dynamic ditelluride exchange across chemically and structurally diverse ditellurides, we further explored the potential mechanism of the ditelluride‐ditelluride exchange. The choice of solvent appears to influence the position of the NMR signals as shown in Figures S11 and S12. Performing the Te─Te exchange reaction between Ph_2_Te_2_ and (*p‐*MePh)_2_Te_2_ in solvents such as DMSO‐d6, acetone‐d6, and CDCl_3_ resulted in shifts in peak positions, both for reactants and products. However, no other significant changes in equilibrium or reaction rate were observed under these conditions, because a full equilibrium was reached within 20 min at room temperature (Figures S11‐S12). The shifts in ^125^Te NMR peak positions observed in different solvents are likely due to variations in solvent polarity and specific interactions with the tellurium species. Despite these shifts, the underlying equilibrium remains unaffected, proving that solvent effects are limited to spectral properties. Moreover, the effect of temperature was also assessed by conducting the reaction at ‐50 °C in CDCl_3_ (Figure S13). The reaction rate did not noticeably slow down, and while the NMR signals shifted, the overall equilibrium remained unaffected within 20 min.

To investigate whether the Ph_2_Te_2_ and (*p‐*MePh)_2_Te_2_ exchange reaction is light‐mediated, a brown NMR tube was employed to shield the reaction mixture from light exposure. No significant difference was found between the visible light‐exposed systems and the system deprived of visible light, indicating that the ditelluride‐ditelluride exchange is not a photochemically‐driven process, implying that the reaction likely does not occur via a radical mechanism (Figure S14). Additionally, the effect of radical scavengers was evaluated by introducing 2 equivalents of 2,2,6,6‐tetramethylpiperidine 1‐oxyl (TEMPO) to Ph_2_Te_2_ and (*p‐*MePh)_2_Te_2_ before the equilibrium could be reached (Figure S15). However, the presence of TEMPO did not prevent the establishment of equilibrium within 20 min at room temperature, again indicating that the reaction does not primarily proceed through a radical mechanism. These findings indicate that the ditelluride‐ditelluride exchange process remains unaffected by solvent polarity, temperature changes, absence of visible light, and presence of radical scavengers, highlighting that it follows a stable and efficient equilibrium‐driven mechanism without an external stimulus.

**Figure 6:**
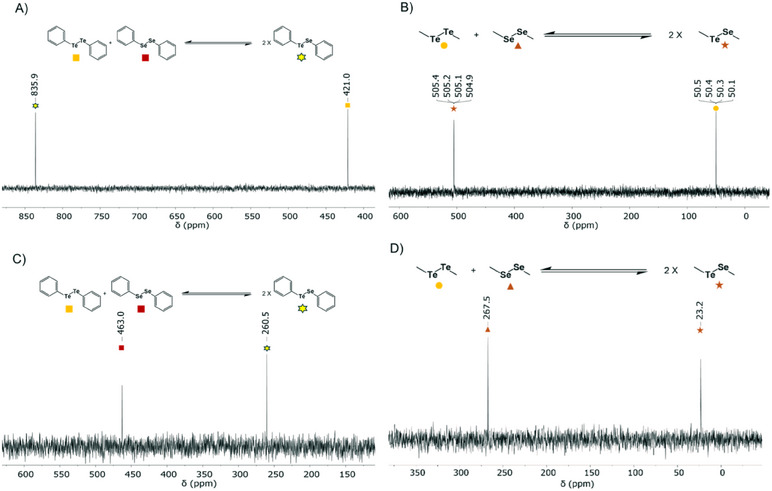
^125^Te NMR and ^77^Se NMR spectra of ditelluride‐diselenide systems containing both aliphatic and aromatic substituents, forming equilibrium upon prolonged incubation at room temperature in CDCl_3_. A) ^125^Te NMR spectrum of a molar equivalent mixture of Ph_2_Te_2_ and Ph_2_Se_2_. B) ^125^Te NMR spectrum of a molar equivalent mixture of Me_2_Te_2_ and Me_2_Se_2_. C) ^77^Se NMR spectrum of a molar equivalent mixture of Ph_2_Te_2_ and Ph_2_Se_2_. D) ^77^Se NMR spectrum of a molar equivalent mixture of Me_2_Te_2_ and Me_2_Se_2_.

Having shown that ditelluride systems can rapidly reach equilibrium, showing their potential for forming dynamic combinatorial libraries, we next explored the dynamic exchange between ditellurides and related diselenides. We first investigated whether a heterogeneous mixture of ditelluride‐diselenide system was able to equilibrate at a comparable rate as a homogenous ditelluride system. However, the reaction between equimolar amounts of Ph_2_Te_2_ and Ph_2_Se_2_ showed that the exchange process of the heterogeneous ditelluride‐diselenide mixture was slower than in the sole ditelluride system (Figure 6A). The time course of ditelluride‐diselenide exchange showed that equilibrium in the heterogeneous system was achieved gradually over several hours, both in the presence and absence of visible light (Figures S16–S17), in contrast to studies that claimed that the Te─Te/Se─Se exchange does not occur under dark conditions, although we may not rule out that distinct mechanisms can take place depending on the exact ditelluride system and conditions used.^[^ ^11^ ^]^ The time‐course experiments showed a clear trend that the aromatic mixtures of ditellurides and diselenides reached an equilibrium faster than the aliphatic counterparts (Figure S16). The reactivity can be ranked in the order of Ph_2_Te_2_ + Ph_2_Se_2_ > Ph_2_Te_2_ + Me_2_Se_2_ > Me_2_Te_2_ + Me_2_Se_2_. For the aromatic mixtures, the equilibrium was reached already after 5 h (Figure S16). Although much slower than the aromatic Te‐Te exchange, it stayed consistent and did not change significantly throughout a period of 24 h. The Ph_2_Te_2_ + Me_2_Se_2_ equilibrium was reached after ≈18 h (Figure S16). Instead of the steep incline seen in the beginning Ph_2_Te_2_ + Ph_2_Se_2_, it showed a steady increase in equilibrium product PhTeSeMe. After ≈65 h, equilibrium was finally reached for the aliphatic system Me_2_Te_2_ + Me_2_Se_2_ (Figure S16). In Figure 6, the final equilibrium is shown in both ^125^Te NMR and ^77^Se NMR spectra, where only two of three components can be seen at a time, due to their different heteroatoms. The addition of (*p‐*MePh)_2_Te_2_ to the preestablished equilibrium of Ph_2_Te_2_ and Ph_2_Se_2_ quickly created a new three‐way equilibrium, more rapidly than the initial one, suggesting that ditellurides expedite the process, even when the slower diselenides are involved (Figure S18).

**Figure 7:**
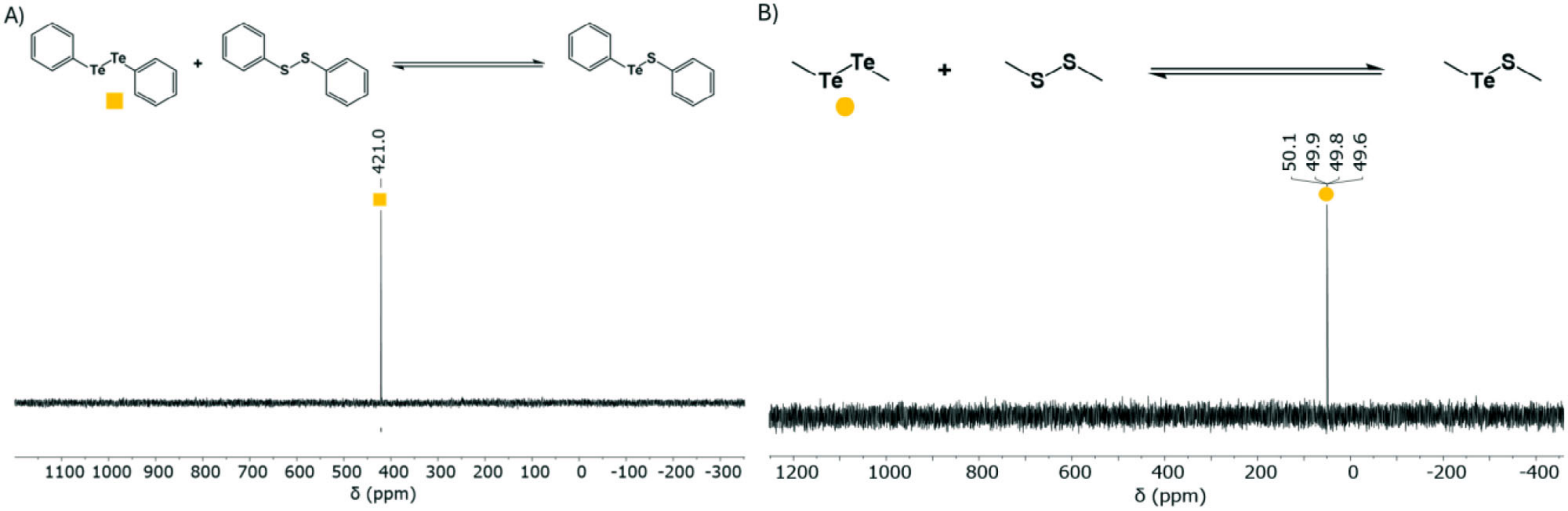
^125^Te NMR spectra of ditelluride‐disulfide systems containing aromatic and aliphatic substituents, showing no equilibrium. A) ^125^Te NMR spectrum of a molar equivalent mixture of Ph_2_Te_2_ and Ph_2_S_2_ after 120 h at room temperature. B) ^125^Te NMR spectrum of a molar equivalent mixture of Me_2_Te_2_ and Me_2_S_2_ after 94 h at room temperature.

To finalize the exploration between dichalcogen‐dichacogen bonds involving ditellurides, we also investigated the potential equilibrium between aromatic Ph_2_Te_2_ and Ph_2_S_2_, and aliphatic Me_2_Te_2_ and Me_2_S_2_. Expecting an equilibrium to form, like the Te‐Se system, we did not observe any new Te species upon incubation for several days at room temperature in CDCl_3_ (Figure 7). Despite prolonged reaction and regular monitoring, no equilibrium was detected between the two species for both aromatic and aliphatic systems under our conditions (Figure S19). To attempt the ditelluride‐disulfide exchange reaction to proceed, the same two systems were further examined in the presence of catalytic amounts of thiophenol, because of its higher reactivity to potentially enable the formation of a dynamic equilibrium. However, this approach did not result in the formation of the proposed equilibrium products PhTeSPh and MeTeSMe, respectively, which should have a downfield chemical shift (PhTeSPh at ≈1000 ppm) (Figure S20).^[^ ^12^ ^]^

**Figure 8:**
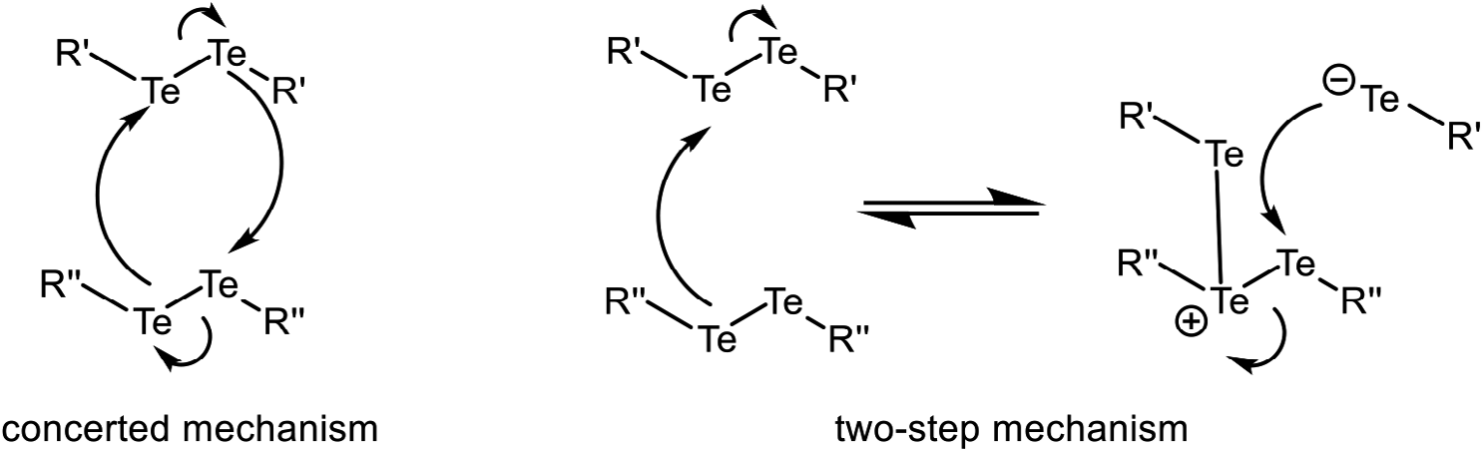
Probing the mechanism of the ditelluride exchange.

With the aim of understanding the mechanism behind the above‐discussed 1:1 equilibrium of diaryl ditellurides, a dispersion‐corrected density functional theory (DFT) analysis has been performed at the ZORA‐BLYP‐D3(BJ)/TZ2P level of theory in chloroform (Table S2). We have first considered the equilibrium between diaryl ditelluride and *para*‐methyl diaryl ditelluride. Both concerted and two‐step mechanisms have been considered (Figure 8). In the equilibrium between diaryl ditelluride and *para*‐methyl diaryl ditelluride, the transition state corresponds to that of a non‐synchronous concerted mechanism involving the lone pair of the tellurium. This non‐synchronous concerted mechanism is supported by the longer Te─Te bond that holds the σ^∗^ LUMO, whereas the fragment with the shorter Te─Te bond holds the π^*^ HOMO (Figure S21). In this way, the inter‐bond lobes of the σ* better fit with the lobes of the π* fragment, together with the fact that this σ* is at lower energy while the π* is at higher energy. The free energy barrier amounts to 17.8 kcal mol^−1^, whereas the reaction free energy amounts to only −3.5 kcal mol^−1^ (Table 1).

**Table 1:** Enthalpies (∆*H*) and Gibbs free (∆*G*) activation and reaction energies (in kcal mol^−1^) of model exchange reactions.

	∆*E* ^≠^		∆*E* ^r^	
Equilibrium	∆*H*	∆*G*	∆*H*	∆*G*
Ph_2_Te_2_ + (*p‐*MePh)_2_Te_2_ ↔ 2 PhTeTe(*p‐*MePh)	8.0	17.8	1.4	−3.5
Ph_2_Te_2_ + Ph_2_Se_2_ ↔ 2 PhTeSePh	12.8	20.7	−0.1	−2.2
Ph_2_Te_2_ + Ph_2_S_2_ ↔ 2 PhTeSPh	17.8	26.5	1.3	−2.2

[a] Concerted mechanism. Computed at ZORA‐BLYP‐D3(BJ)/TZ2P using COSMO to simulate solvation in chloroform.

**Figure 9:**
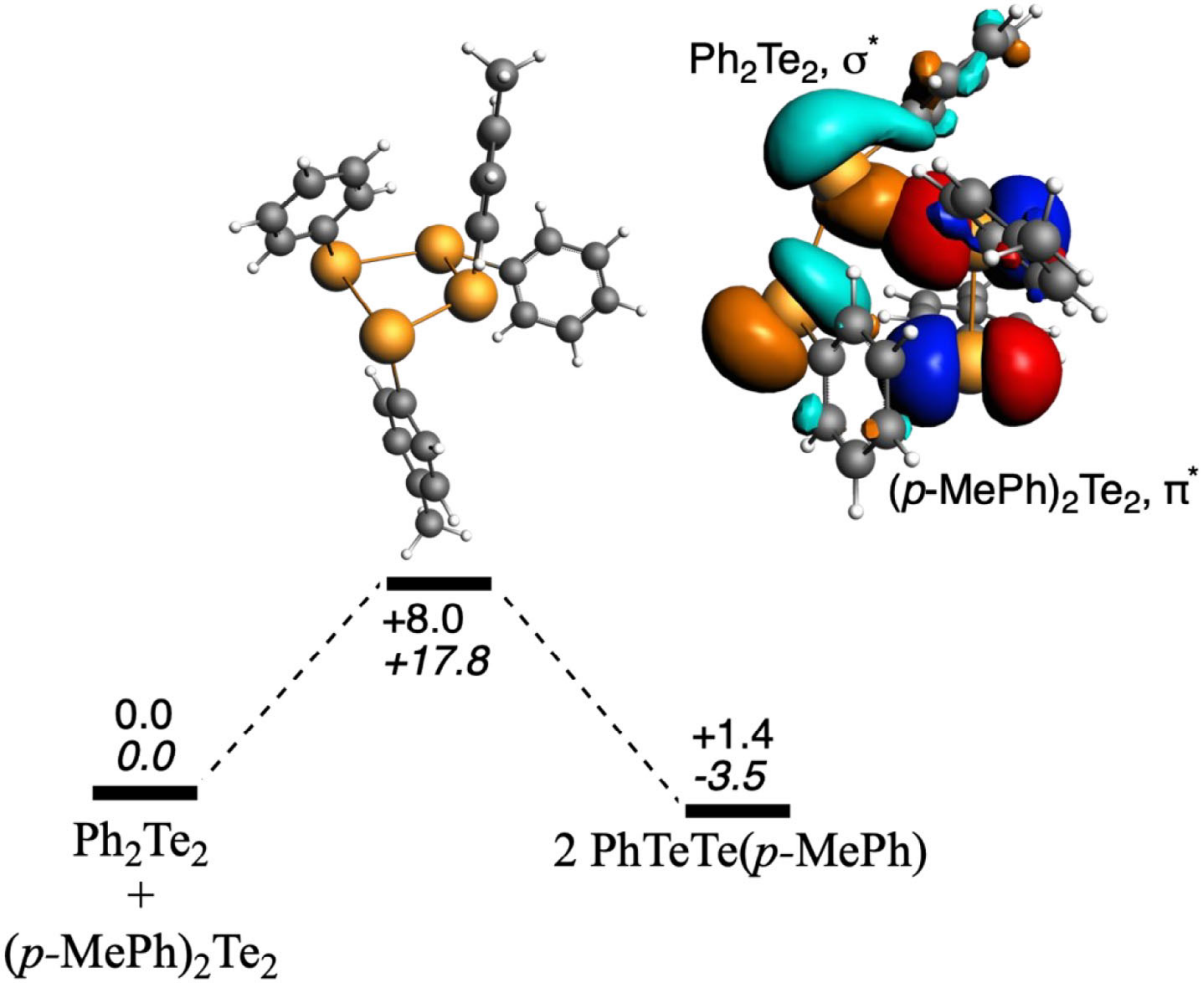
Computed reaction profile for the equilibrium between diaryl ditelluride and *para*‐methyl diaryl ditelluride. Enthalpies and Gibbs (in italics) energies are enclosed (in kcal mol^−1^). The isosurfaces of the superposed HOMO and LUMO of the two fragments to build the TS (isovalue = 0.03) are also enclosed. Computed at ZORA‐BLYP‐D3(BJ)/TZ2P in chloroform.

The proposed non‐synchronous concerted mechanism agrees well with the above experimental data regarding the instant equilibrium in this ditelluride exchange. First, all explorations to find the stepwise process yield the concerted pathway, involving the lone pair of the chalcogen (Figure 9). Second, the fact that a radical mechanism has not been found either also agrees with the finding that the referred ditelluride equilibrium is not a photochemically‐driven process (*vide supra*). In particular, the dissociation of the Te─Te bond, followed by a radical attack on another Te─Te bond, and the follow‐up dissociation would involve a barrier of the order of the bond dissociation energy of Ph_2_Te_2_, which is not viable (Table S3).

For the Ph_2_Te_2_‐Ph_2_Se_2_ exchange, the barrier increases up to 20.7 kcal mol^−1^ (Table 1), thus supporting the expected slower exchange process of this heterogeneous ditelluride‐diselenide mixture. Also, in this case, the mechanism is likely non‐synchronous concerted, supported by the fact that light is not required, as stated above. Finally, in the case of the Ph_2_Te_2_‐Ph_2_S_2_ exchange, the barrier further increases up to 26.5 kcal mol^−1^ (Figures S21 and S22). Thus, the computed barriers agree well with the above‐presented experimental data showing the slower exchange between ditellurides and diselenides, and non‐observable exchange with disulfides. This increase in the barrier should also expect an increase in the chalcogen‐chalcogen bond strength from Te─Te to Se─Se and to S─S. However, this is not observed due to the increase of strain energy between the phenyl groups when going from Te to Se and to S, thus making the bond strengths very similar (Table S3). At difference, the HOMO–LUMO gaps do support the higher barrier, by increasing in the same direction from ‐2.3 to ‐2.8 and ‐3.5 kcal mol^−1^, which is a factor that weakens the stabilizing HOMO–LUMO interaction between the reactants (Figure S23).^[^ ^13^ ^]^

## 3 Conclusions

The dynamic exchange of covalent bonds has been central to the area of dynamic combinatorial chemistry and its numerous applications in supramolecular chemistry, material science, and medicinal chemistry. While the chemistry of disulfide‐disulfide exchange and diselenide‐diselenide exchange has been comprehensively studied, the related ditelluride‐ditelluride exchange has remained poorly understood. Using ^125^Te NMR spectroscopy, we have demonstrated that the ditelluride‐ditelluride exchange between chemically and structurally diverse diaryl/dialkyl ditellurides, including sterically demanding systems, proceeds very rapidly and produces the 1:1 mixture of reactants and products. In contrast to the disulfide‐disulfide exchange and diselenide‐diselenide exchange that might require an external stimulus for the reactions to progress, the exceptionally efficient ditelluride‐ditelluride exchange does not require any special condition or external stimulus to proceed. Our studies demonstrate that the ditelluride‐ditelluride exchange can be carried out in various solvents, at very low temperatures, in the absence of visible light, and in the presence of radical scavengers. Synergistic experimental and computational studies suggest that the ditelluride‐ditelluride exchange does not proceed via a radical mechanism, but rather via a mechanistically distinct non‐synchronous concerted mechanism in which Te‐Te bonds are broken and formed simultaneously. Combined NMR spectroscopic and quantum chemical analyses have, furthermore, shown that the efficiency of the chalcogen‐chalcogen exchange involving ditellurides follows the trend Te‐Te > Te‐Se > Te‐S. Taken together, these results highlight that ditellurides might serve as excellent dynamic systems that may find widespread applications across chemical sciences.

## 4 Experimental Section

### Synthesis of Diaryl Ditellurides

t‐Butyllithium (1.6–3.4 mL, 1.6–3.4 mmol) was dropwise added to a stirred solution of para‐substituted aryl halides (0.13–0.50 g, 0.53–2.3 mmol) in anh. THF at ‐78 °C in an oven‐dried, argon‐filled microwave vial. After 1 h, the cooling was removed, and the reaction mixture was allowed to warm to room temperature. Then, tellurium (200 mesh, 0.16–0.35 g, 1.3–2.8 mmol) was added to the solution, ensuring the flask was purged of air using argon. The suspension was stirred for 1 h at room temperature and then quenched using ice‐cold methanol (4–10 mL). The reaction mixture was concentrated *in vacuo*, and purified using column chromatography using appropriate mixtures of DCM in PE (0‐100%), to yield diaryl ditellurides as red solids.

1,2‐bis(4‐methoxyphenyl)ditellane. Red solid (249 mg, 50%); mp 55–57 °C; ^1^H NMR (500 MHz, CDCl_3_) δ 7.70 (dt, *J* = 8.8, 2.2 Hz, 4H), 6.75 (dt, *J* = 8.8, 2.1 Hz, 4H), 3.80 (s, 6H); ^13^C NMR (126 MHz, CDCl_3_) δ 160.3, 140.4, 115.2, 97.7, 55.3; ^125^Te NMR (158 MHz, CDCl_3_) δ 458.2.

1,2‐di‐*p‐*tolylditellane. Red solid (373 mg, 74%); mp 50–52 °C; ^1^H NMR (500 MHz, CDCl_3_) δ 7.68 (dt, *J* = 8.0, 1.6 Hz, 4H), 7.01 (dt, *J* = 7.8 Hz, 4H), 2.38 (s, 6H); ^13^C NMR (126 MHz, CDCl_3_) δ 138.2, 130.3, 104.2, 21.2.^125^Te NMR (158 MHz, CDCl_3_) δ 427.8.

1,2‐bis(4‐chlorophenyl)ditellane. Dark red solid (139 mg, 28%); mp 109–112 °C; ^1^H NMR (500 MHz, CDCl_3_) δ 7.69 (dt, *J* = 8.1, 1.7 Hz, 4H), 7.16 (dt, *J* = 8.2, 1.6 Hz, 4H). ^13^C NMR (126 MHz, CDCl_3_) δ 139.3, 135.0, 129.6, 105.3. ^125^Te NMR (158 MHz, CDCl_3_) δ 447.2.

1,2‐bis(4‐(trifluoromethyl)phenyl)ditellane. Light red solid (323 mg, 64%); mp 79–81 °C; ^1^H NMR (500 MHz, CDCl_3_) δ 7.90 (d, *J* = 8.0 Hz, 4H), 7.43 (d, *J* = 7.9 Hz, 4H); ^13^C NMR (126 MHz, CDCl_3_) δ 137.5, 126.1; ^19^F NMR (471 MHz, CDCl_3_) δ ‐62.7; ^125^Te NMR (158 MHz, CDCl_3_) δ 428.8.

1,2‐bis(2,6‐dimethylphenyl)ditellane. Brown solid (112 mg, 22%); mp 146–148 °C; ^1^H NMR (500 MHz, CDCl_3_) δ 7.11 – 7.01 (m, 6H), 2.38 (d, *J* = 6.0 Hz, 12H). ^13^C NMR (126 MHz, CDCl_3_) δ 146.3, 129.6, 126.0, 115.9, 30.3. ^125^Te NMR (158 MHz, CDCl_3_) δ 205.5.

1,2‐bis(2,6‐diisopropylphenyl)ditellane. Brown solid (147 mg, 37%); mp 112–113 °C; ^1^H NMR (500 MHz, CDCl_3_) δ 7.18 (t, *J* = 7.6 Hz, 2H), 7.04 (dd, *J* = 7.6, 0.8 Hz, 4H), 3.52 (hept, *J* = 6.8 Hz, 4H), 1.03 (dd, *J* = 6.8, 0.9 Hz, 24H); ^13^C NMR (126 MHz, CDCl_3_) δ 155.8, 130.3, 122.9, 117.3, 40.5, 24.2; ^125^Te NMR (158 MHz, CDCl_3_) δ 191.0.

1,2‐di(naphthalen‐1‐yl)ditellane. Red solid (332 mg, 64%); mp 117–120 °C; ^1^H NMR (500 MHz, CDCl_3_) δ 8.14 (dd, *J* = 7.1, 1.2 Hz, 1H), 8.01 (dq, *J* = 8.4, 0.9 Hz, 1H), 7.79 (ddt, *J* = 18.2, 8.2, 0.9 Hz, 2H), 7.46 (ddd, *J* = 8.1, 6.8, 1.2 Hz, 1H), 7.33 (ddd, *J* = 8.3, 6.8, 1.3 Hz, 1H), 7.22 (dd, *J* = 8.2, 7.1 Hz, 1H); ^13^C NMR (126 MHz, CDCl_3_) δ 140.7, 136.2, 133.5, 133.0, 130.2, 128.9, 126.9, 126.4, 126.3, 111.4.; ^125^Te NMR (158 MHz, CDCl_3_) δ 336.9.

1,2‐diethylditellane. Red liquid (147 mg, 39%); ^1^H NMR (500 MHz, CDCl3) δ 3.04 (dq, J = 7.6, 1.2 Hz, 2H), 1.62 (td, J = 7.6, 1.2 Hz, 3H). ^13^C NMR (126 MHz, CDCl3) δ 19.8, ‐4.4. ^125^Te NMR (158 MHz, CDCl_3_) δ 166.6 (q, *J* = 22.3 Hz).

1,2‐di‐tert‐butylditellane. Red Liquid (302 mg, 91%); ^1^H NMR (500 MHz, CDCl3) δ 1.66 (s, 18H); ^13^C NMR (126 MHz, CDCl3) δ 36.5, 23.1; ^125^Te NMR (158 MHz, CDCl3) δ 477.9 (dh, *J* = 47.0, 20.7, 20.3 Hz).

### Synthesis of Dialkyl Ditellurides

Tellurium (200 mesh, 334–408 mg, 2.6–3.2 mmol) was suspended in anhydrous THF in an argon‐purged, oven‐dried microwave vial and cooled to 0 °C. Alkyl lithium (1.9–2.0 mL, 3.1–3.3 mmol) was added dropwise to the reaction mixture, then allowed to warm to room temperature and stirred for 1 h. A saturated aqueous solution of NH_4_Cl was then added to the reaction and extracted using EtOAc. The combined organic phase was dried over MgSO_4_, and activated charcoal was added before being filtered and concentrated under reduced pressure to give a deep red liquid.

### Acquisition of ^125^Te and ^77^Se NMR Spectra

^125^Te NMR and ^77^Se NMR spectra were recorded on a 500 MHz JEOL RESONANCE ECZ500R NMR spectrometer. Deuterated chloroform was used as solvent unless otherwise stated. Stock solutions were prepared from purified solids or oils and dissolved in CDCl_3_ with a concentration standardized to be 50 or 100 mM. 100 mM stocks were used in systems with more than two components. Diphenyl ditelluride was used as an internal standard at 421.0 ppm. Samples were prepared in 5 mm NMR tubes and spectra were recorded at room temperature. The ^125^Te NMR spectra were recorded at 157.7 MHz with an acquisition time of 0.115 s and a spectral width for data acquisition of 192 kHz. The data were collected for 16k transients, with a pulse width of 7.25 µs and a relaxation delay of 1.5 s. The FIDs were transformed with an exponential line broadening function of 15−50 Hz. ^125^Te NMR spectra were recorded using 512 scans, while ^77^Se NMR spectra were recorded using 1024 scans.

### Quantum Chemical Analysis

All DFT calculations were performed with the Amsterdam Density Functional (ADF) program using relativistic, dispersion‐corrected density functional theory (DFT) at the ZORA‐BLYPD3BJ/TZP level of theory for geometry optimizations and energy calculations, with the full electron model for all atoms (no frozen core).^[^ ^14^ ^]^ Solvation in chloroform was simulated using the conductor‐like screening model (COSMO). All stationary points were verified through vibrational analysis to be minima (0 imaginary frequencies) or transition states (1 imaginary frequency) on the potential energy surface.

## Conflict of Interest

The authors declare no conflict of interest.

## Supporting information



Supporting Information

## Data Availability

The data that support the findings of this study are available in the supplementary material of this article.
