# Supplementary material for: Dynamic Combinatorial Chemistry of Ditellurides
Source: Chemistry. 2025 May 28;31(35):e202501291. doi: 10.1002/chem.202501291 (PMC12188154; doi:10.1002/chem.202501291)
Supplement: Supplementary file 1 — Supporting Information [file CHEM-31-e202501291-s001.pdf]

Supporting Information

Dynamic Combinatorial Chemistry of Ditellurides

Christian D. Fisker,[a] Jordi Poater,[b,c] F. Matthias Bickelhaupt,[d,e,f]* and Jasmin Mecinović[a]*

[a] Department of Physics, Chemistry and Pharmacy, University of Southern Denmark, Campusvej 55, 5230 Odense, Denmark
E-mail: mecinovic@sdu.dk

[b] ICREA, Passeig Lluís Companys 23, 08010 Barcelona, Spain

[c] Departament de Química Inorgànica i Orgànica & IQTCUB, Universitat de Barcelona, Martí i Franquès 1-11, 08028 Barcelona, Spain

[d] Department of Chemistry and Pharmaceutical Sciences, Amsterdam Institute of Molecular and Life Sciences (AIMMS), Vrije Universiteit Amsterdam, De Boelelaan 1108, 1081 HZ Amsterdam, The Netherlands
E-mail: f.m.bickelhaupt@vu.nl

[e] Institute for Molecules and Materials, Radboud University, Heyendaalseweg 135, 6525 AJ Nijmegen, The Netherlands

[f] Department of Chemical Sciences, University of Johannesburg, Auckland Park, Johannesburg 2006, South Africa

Table of Contents

S1 Overview of compounds ... 3

S2 Synthesis schemes ... 6

S3 Ditelluride Dynamic Combinatorial Libraries ... 7

S3.1 Two compound equilibrium of diaryl ditellurides. ... 7

S3.2 Three-compound equilibrium of diaryl ditellurides. ... 9

S3.3 Four-compound equilibrium of diaryl ditellurides ... 10

S3.4 Diaryl ditelluride equilibrium using different deuterated solvents ... 14

S3.5 Diaryl ditelluride equilibrium at -50 °C ... 15

S3.6 Diaryl ditelluride equilibrium in the absence of light ... 16

S3.7 Diaryl ditelluride equilibrium with TEMPO radical scavenger ... 17

S4 Ditelluride and diselenide Dynamic Combinatorial Libraries ... 18

S4.1 Time course of equilibrium product formation of ditelluride with diselenides ... 18

S4.2 Ditelluride and diselenide equilibrium formation with the absence of light ... 19

S5 Ditelluride and disulfide Dynamic Combinatorial Libraries ... 20

S5.1 Time course of equilibrium product formation of ditelluride with disulfides ... 20

S5.2 Attempted ditelluride and disulfide equilibrium formation ... 21

S6 Quantum chemical analyses ... 22

S1 Overview of compounds

Table S1. Name, structure, chemical shift in ^{125}Te and ^{77}Se NMR, and marker for each homo- and heteroditellurides, and mixed telluroselenides encountered.

Name	Compound	Chemical shift (ppm)	Marker
Ph_2Te_2	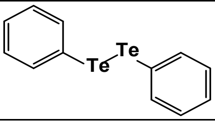	421.0	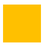
$(p\text{−}MePh)_2Te_2$	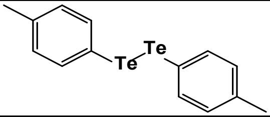	427.8	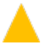
$(p\text{−}MeOPh)_2Te_2$	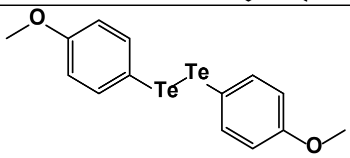	458.1	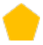
$(p\text{−}CF_3Ph)_2Te_2$	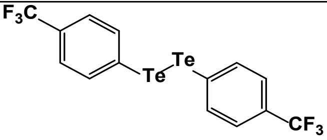	429.3	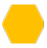
$(p\text{−}ClPh)_2Te_2$	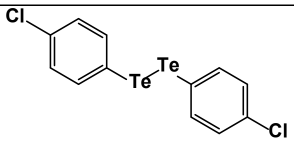	446.8	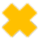
Me_2Te_2	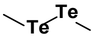	50.1-50.5	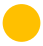
Et_2Te_2	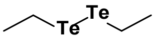	166.4-166.9	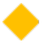
tBu_2Te_2	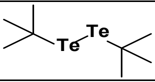	478.4 – 477.4	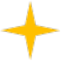
$(o,o\text{−}Me_2Ph)_2Te_2$	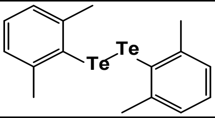	205.5	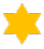
$(o,o\text{−}iPr_2Ph)_2Te_2$	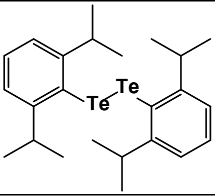	190.9	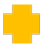
$Naph_2Te_2$	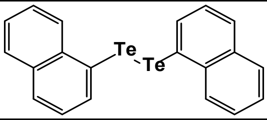	336.9	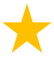
PhTeTe(p−MePh)	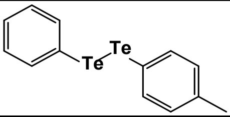	434.7 and 414.1	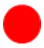

PhTeTe(p−MeOPh)	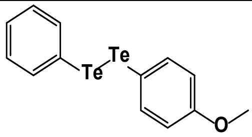	461.6 and 418.5	
PhTeTe(p−CF_3Ph)	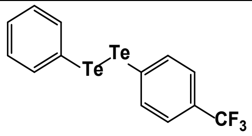	437.6 and 414.8	
PhTeTe(p−ClPh)	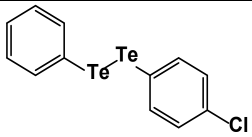	439.1 and 429.3	
(p−CF_3Ph)TeTe(p−MePh)	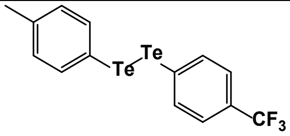	451.2 and 408.5	
(p−CF_3Ph)TeTe(p−MeOPh)	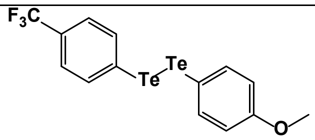	477.2 and 413.5	
(p−MePh)TeTe(p−MeOPh)	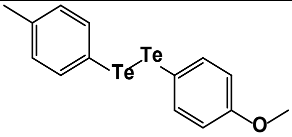	454.3 and 431.9	
(p−ClPh)TeTe(p−MePh)	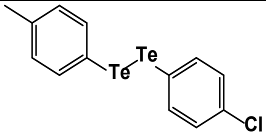	443.0 and 432.7	
(p−ClPh)TeTe(p−MeOPh)	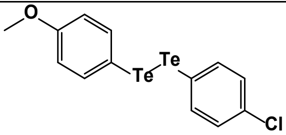	469.2 and 437.7	
(p−MeOPh)TeTe(p−CF_3Ph)	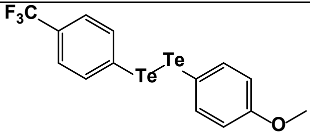	414.0 and 477.7	
(p−ClPh)TeTe(p−CF_3Ph)	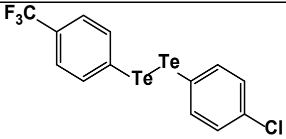	422.0 and 453.9	
MeTeTeEt	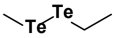	50.1-50.5 and 166.7	
tBuTeTeMe	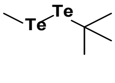	-66.3 - -65.8<br>and<br>598.2-590	
MeTeTePh	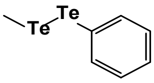	91.9-92.4 and 381.8	

EtTeTePh	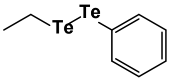	286.0 and 291.0	
PhTeTe(o,o−Me_2Ph)	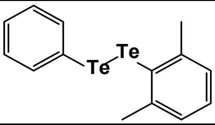	211.3 and 439.2	
PhTeTe(o,o−iPr_2Ph)	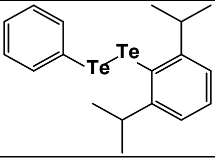	478.8 and 156.3	
(o,o−Me_2Ph)TeTe(o,o−iPr_2Ph)	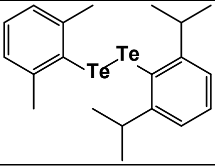	148.7 and 247.3	
NaphTeTe(o,o−iPr_2Ph)	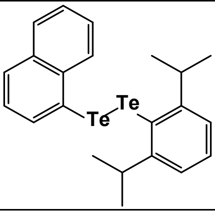	161.8 and 374.8	
Ph_2Se_2	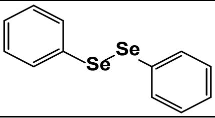	463.3	
Me_2Se_2	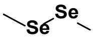	267.3	
PhTeSePh	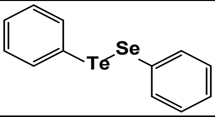	^{125}Te NMR: 835.9<br>^{77}Se NMR: 260.5	
(p−MePh)TeSePh	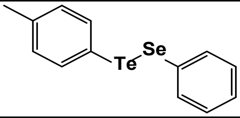	^{125}Te NMR: 835.1<br>^{77}Se NMR: 268.7	
MeTeSeMe	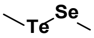	^{125}Te NMR: 504.9-505.4<br>^{77}Se NMR: 23.2	

S2 Synthesis schemes

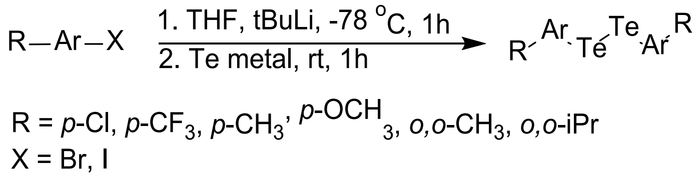

Figure S1. Synthesis scheme of *para*- and *ortho*-substituted diaryl ditelluride compounds.

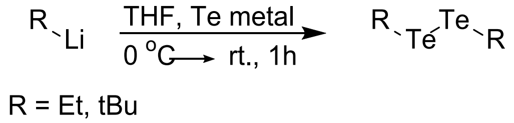

Figure S2. Synthesis scheme dialkyl ditelluride compounds.

S3 Ditelluride Dynamic Combinatorial Libraries

S3.1 Two compound equilibrium of diaryl ditellurides

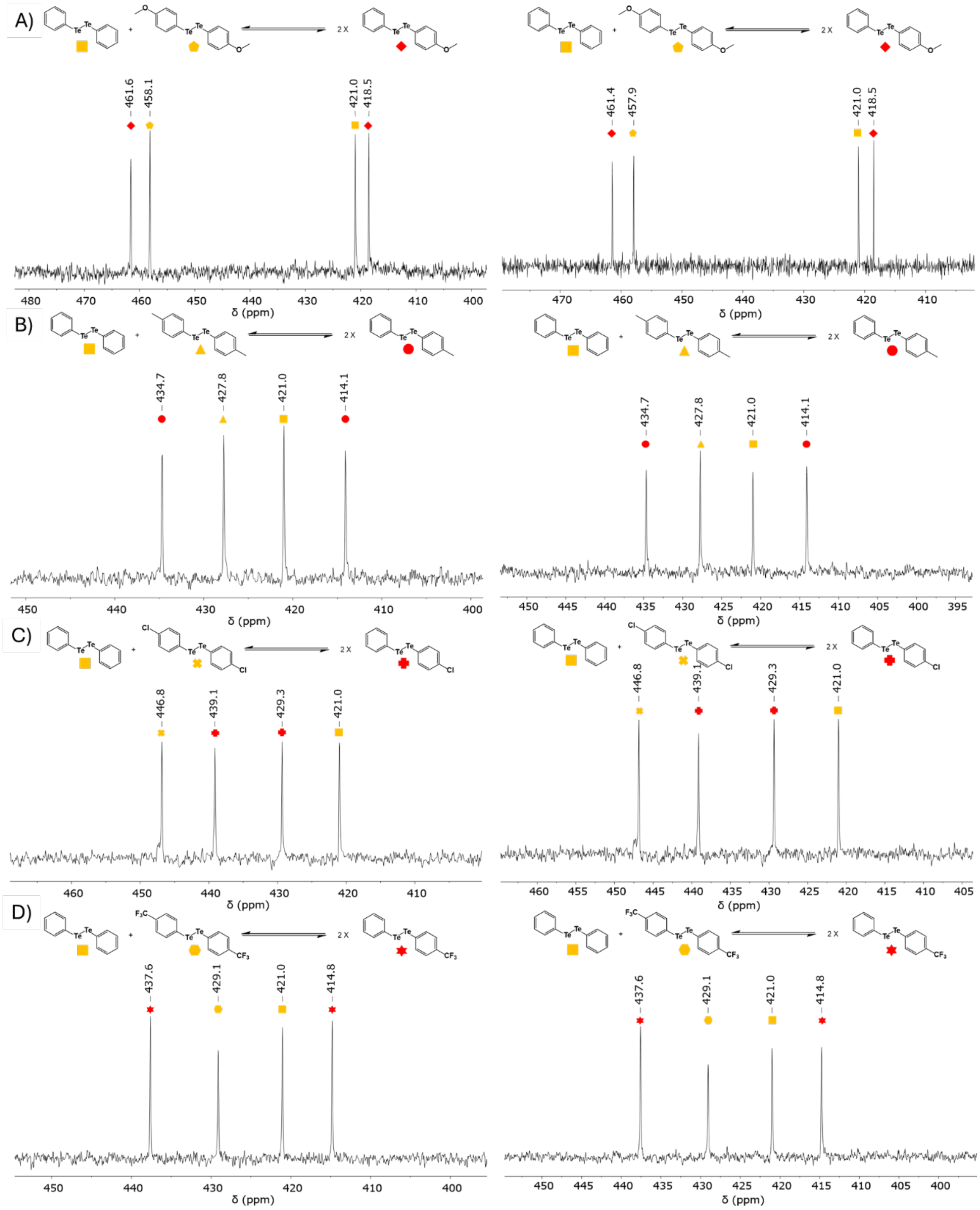

Figure S3. Dynamic combinatorial chemistry of ditellurides. ^{125}Te NMR spectra showing the formation of hetero-ditellurides from the corresponding homo-ditellurides at room temperature in $CDCl_3$. For each combination, spectra are shown within 20 min (left) and overnight (right). A) Ph_2Te_2 and (*p*-MeOPh)$_2Te_2$, B) Ph_2Te_2 and (*p*-MePh)$_2Te_2$, C) Ph_2Te_2 and (*p*-ClPh)$_2Te_2$, D) Ph_2Te_2 and (*p*-CF_3Ph)$_2Te_2$.

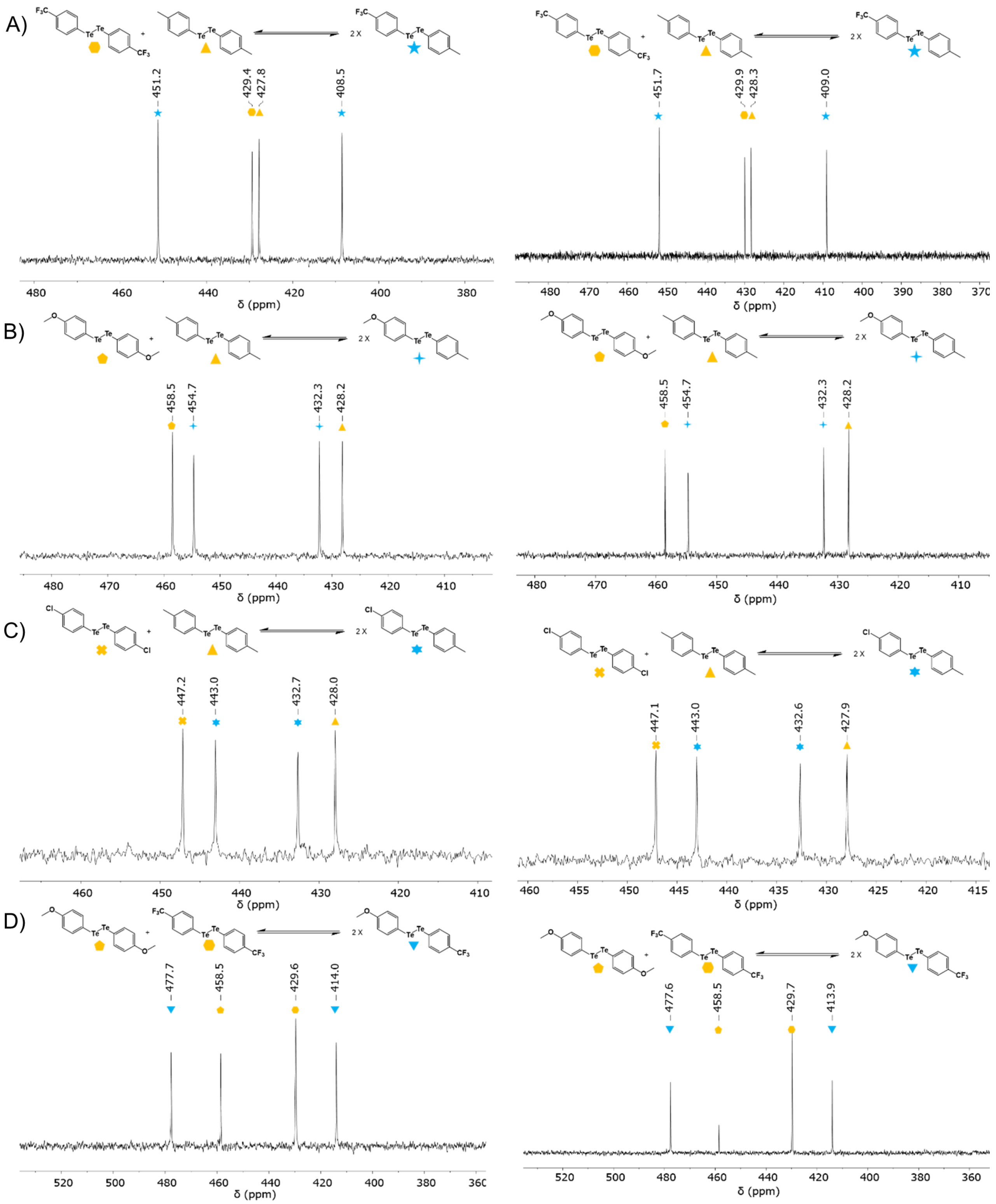

Figure S4. Dynamic combinatorial chemistry of ditellurides (Continued) [125]Te NMR spectra recorded under identical conditions as in Figure S3, within 20 min (left) and overnight (right). A) (p−CF_3Ph)$_2$ Te_2 and (p-MePh)$_2$$Te_2$, B) ($p$-MeOPh)$_2$$Te_2$ and (p-MePh)$_2$$Te_2$, C) ($p$-ClPh)$_2$$Te_2$ and (p-MePh)$_2$$Te_2$, D) ($p$-MeOPh)$_2$$Te_2$ and (p-CF_3Ph)$_2$$Te_2$

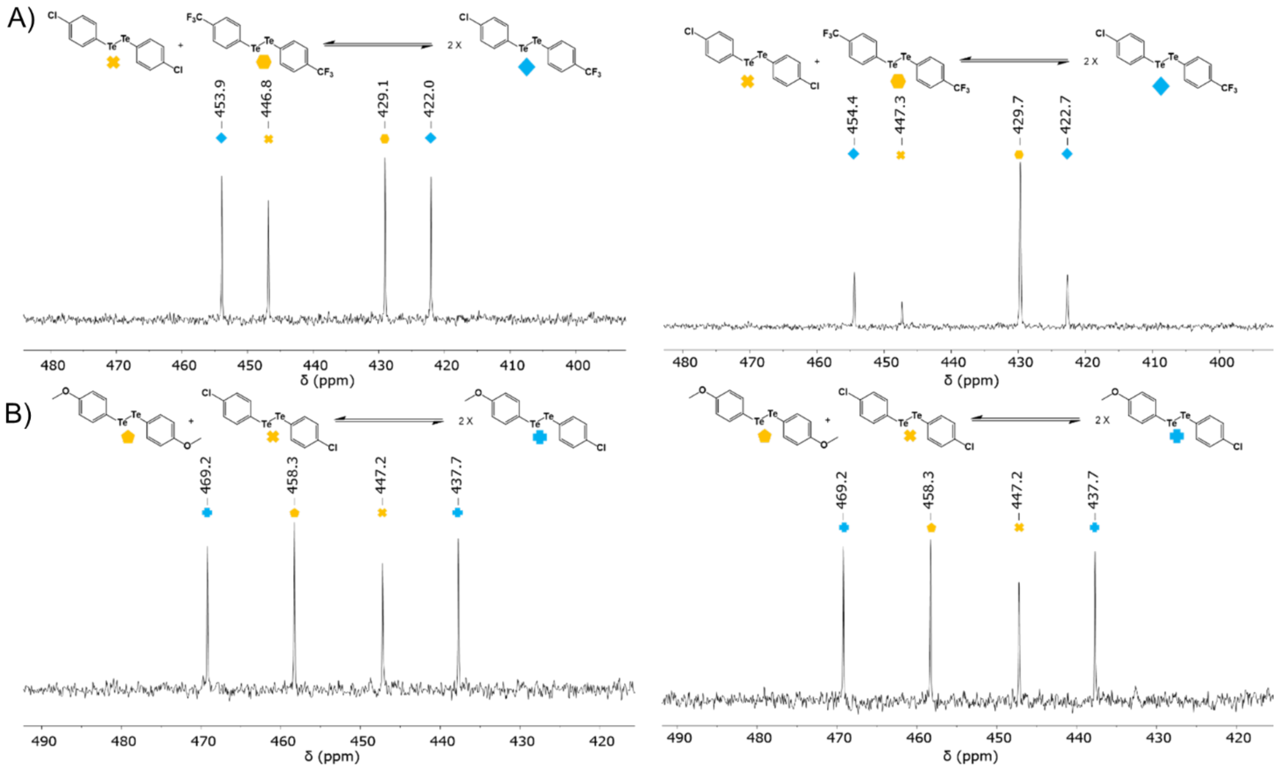

Figure S5. Dynamic combinatorial chemistry of ditellurides (Continued) ^{125}Te NMR spectra recorded under identical conditions as in Figure S3, within 20 min (left) and overnight (right). A) (p-ClPh)$_2$Te$_2$ and (p-CF$_3$Ph)$_2$Te$_2$, B) (p-MeOPh)$_2$Te$_2$ and (p-ClPh)$_2$Te$_2$

S3.2 Three-compound equilibrium of diaryl ditellurides

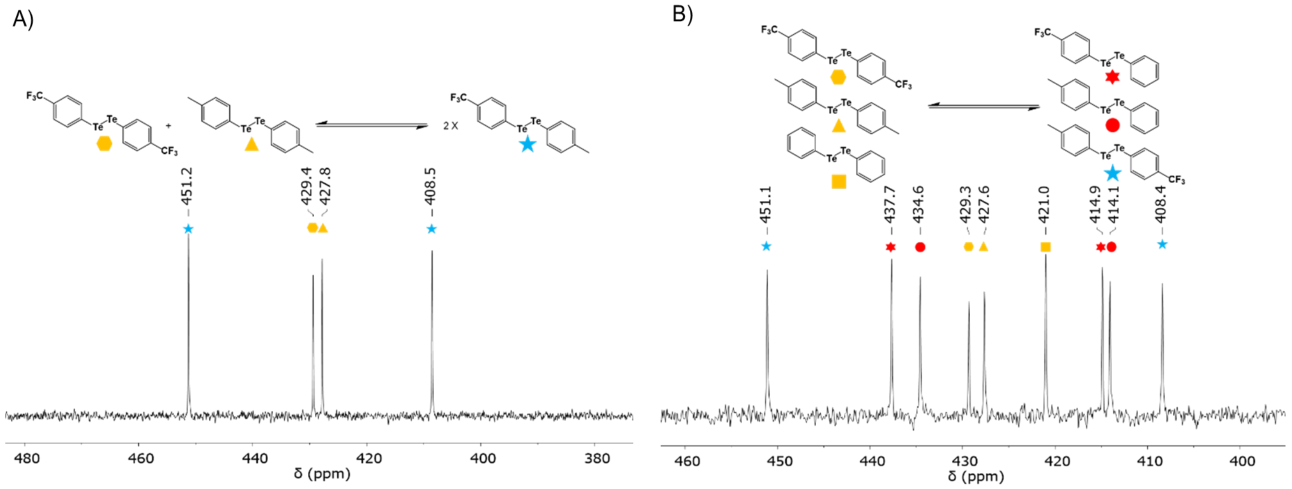

Figure S6. Dynamic combinatorial chemistry of ditellurides. ^{125}Te NMR spectra showing the formation of hetero-ditellurides from the corresponding homo-ditellurides and a new three-component equilibrium upon adding equimolar amounts of Ph$_2$Te$_2$ at room temperature in CDCl$_3$. A) (p–CF$_3$Ph)$_2$Te$_2$ and (p-MePh)$_2$Te$_2$, B) (p–CF$_3$Ph)$_2$Te$_2$, (p-MePh)$_2$Te$_2$, and Ph$_2$Te$_2$.

S3.3 Four-compound equilibrium of diaryl ditellurides

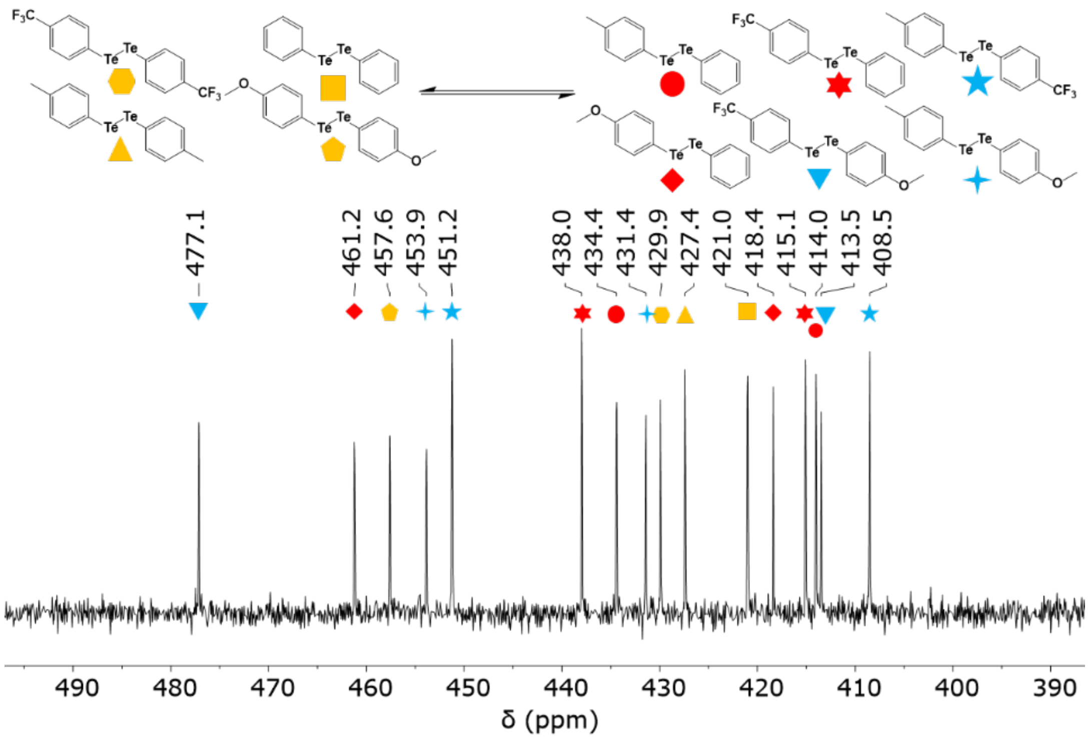

Figure S7. Dynamic combinatorial chemistry of ditellurides. The ^{125}Te NMR spectrum showing the formation of hetero-ditellurides upon mixing (p−MePh)$_2$Te$_2$, (p−CF$_3$Ph)$_2$Te$_2$, Ph$_2$Te$_2$, and (p−OMe Ph)$_2$Te$_2$ at the same time.

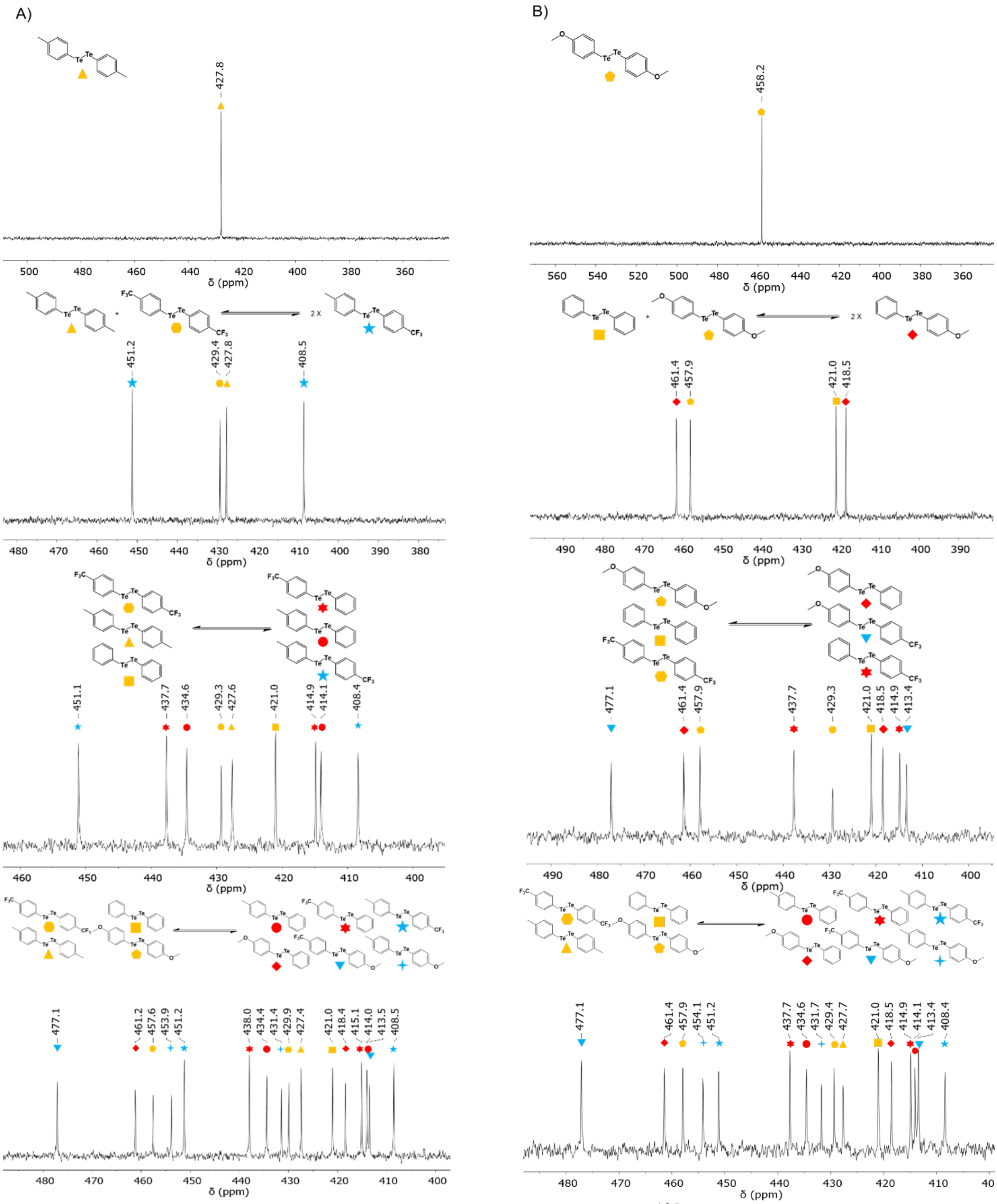

Figure S8. Dynamic combinatorial chemistry of ditellurides. ^{125}Te NMR spectra showing the formation of hetero-ditellurides from the corresponding homo-ditellurides and a new four-component equilibrium upon the sequential addition of ditellurides in $CDCl_3$ to an existing multicomponent equilibrium system. A) Added in order of (p−MePh)$_2$Te$_2$, (p−CF$_3$Ph)$_2$Te$_2$, Ph$_2$Te$_2$, and (p−OMePh)$_2$ Te$_2$. B) Added in order of (p−OMePh)$_2$Te$_2$, Ph$_2$Te$_2$, (p−CF$_3$Ph)$_2$Te$_2$, and (p−MePh)$_2$Te$_2$.

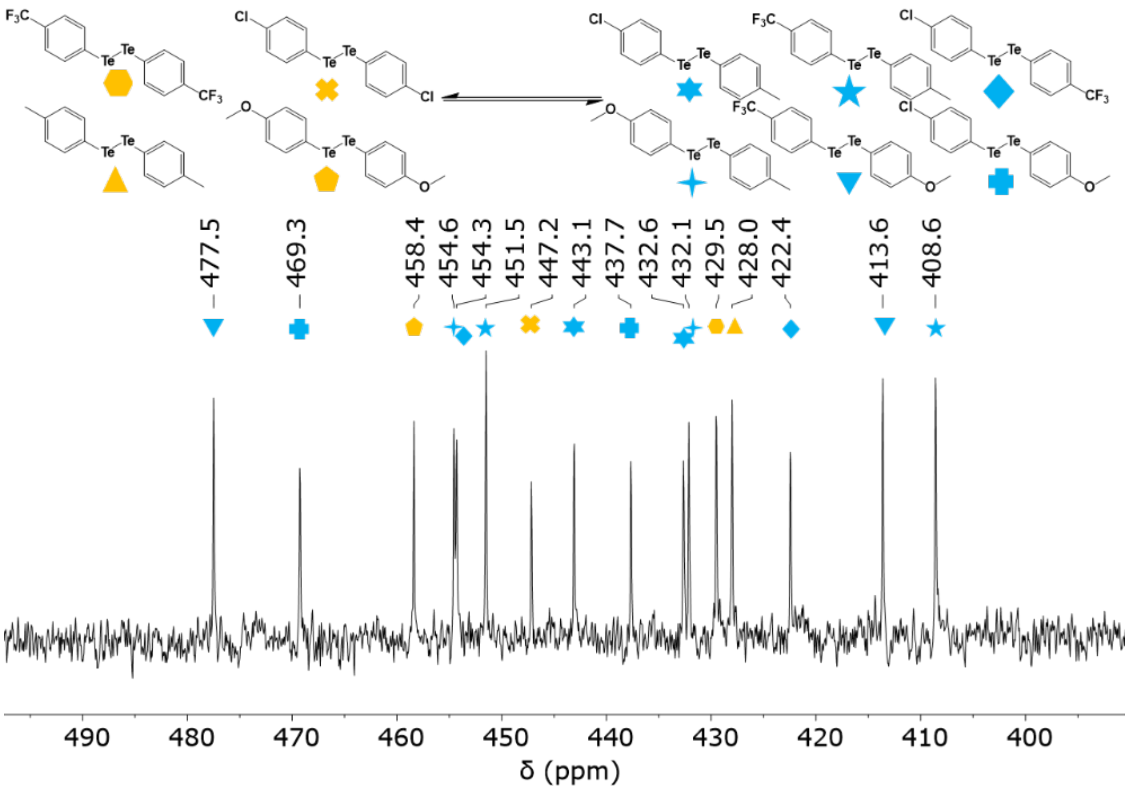

Figure S9. Dynamic combinatorial chemistry of ditellurides. ^{125}Te NMR spectra showing the formation of hetero-ditellurides upon mixing (p−ClPh)$_2$Te$_2$, (p−CF$_3$Ph)$_2$Te$_2$, (p−MePh)$_2$Te$_2$, and (p−OMePh)$_2$Te$_2$ at the same time.

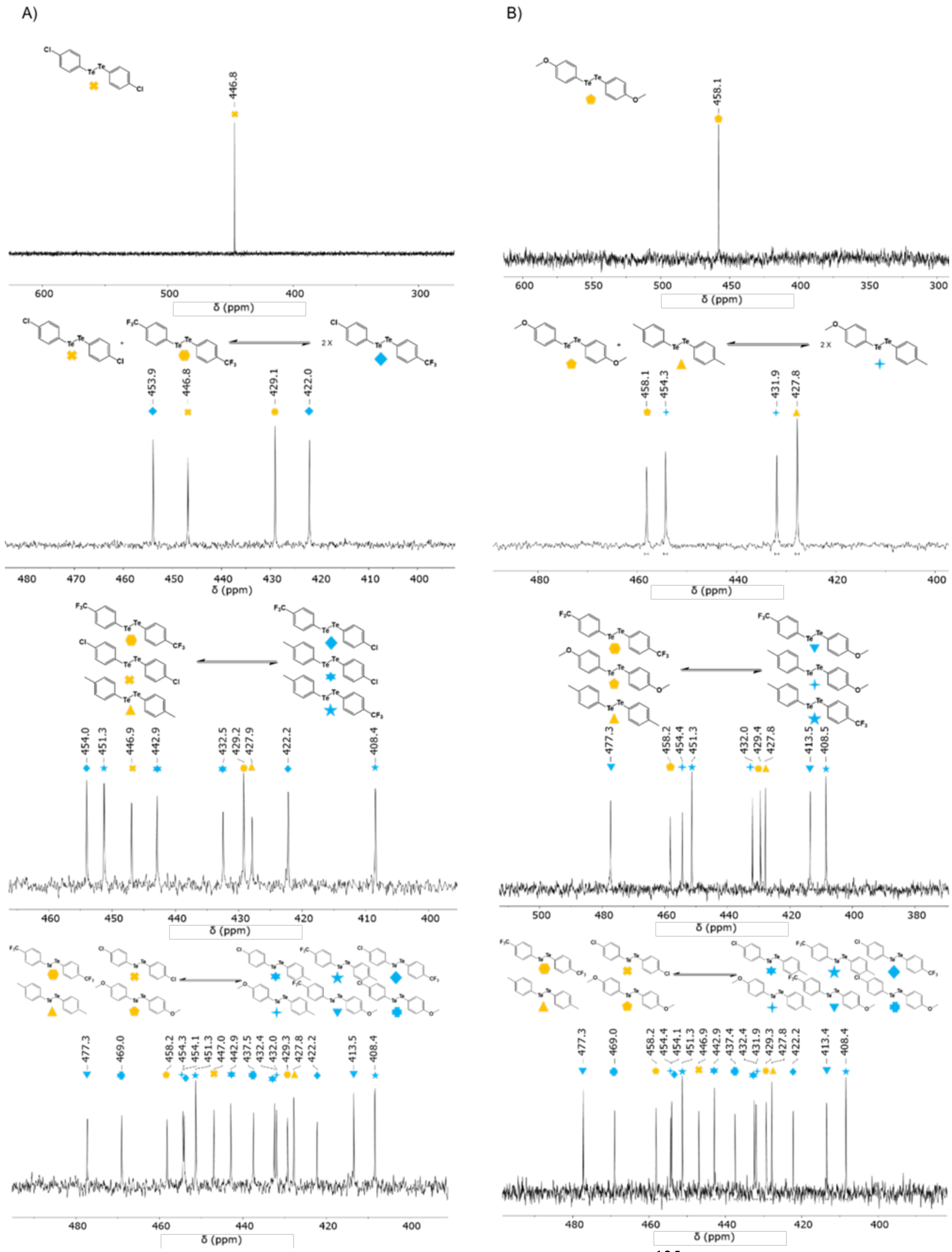

Figure S10. Dynamic combinatorial chemistry of ditellurides. ^{125}Te NMR spectra showing the formation of hetero-ditellurides from the corresponding homo-ditellurides and a new four-component equilibrium upon the sequential addition of ditellurides in $CDCl_3$ to an existing multicomponent equilibrium system in $CDCl_3$. A) Added in order of (*p*−ClPh)$_2$Te$_2$, (*p*−CF$_3$Ph)$_2$Te$_2$, (*p*−MePh)$_2$Te$_2$, and (*p*−OMePh)$_2$Te$_2$. B) Added in order of (*p*−OMePh)$_2$Te$_2$, (*p*−MePh)$_2$Te$_2$, (*p*−CF$_3$Ph)$_2$Te$_2$, and (*p*−ClPh)$_2$Te$_2$.

S3.4 Diaryl ditelluride equilibrium using different deuterated solvents

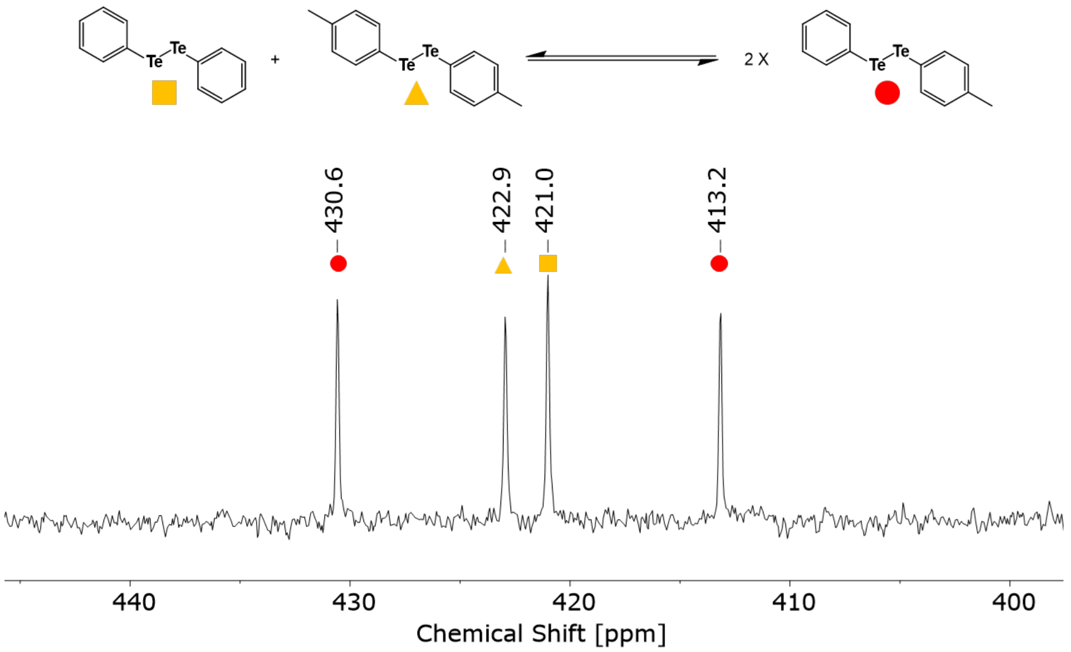

Figure S11. Dynamic Te-Te exchange of ditellurides. The ^{125}Te NMR spectrum showing the formation of the hetero-ditelluride from the corresponding homo-ditellurides by using Ph_2Te_2 and (*p*-MePh)$_2$$Te_2$ within 20 min at room temperature in DMSO-d6.

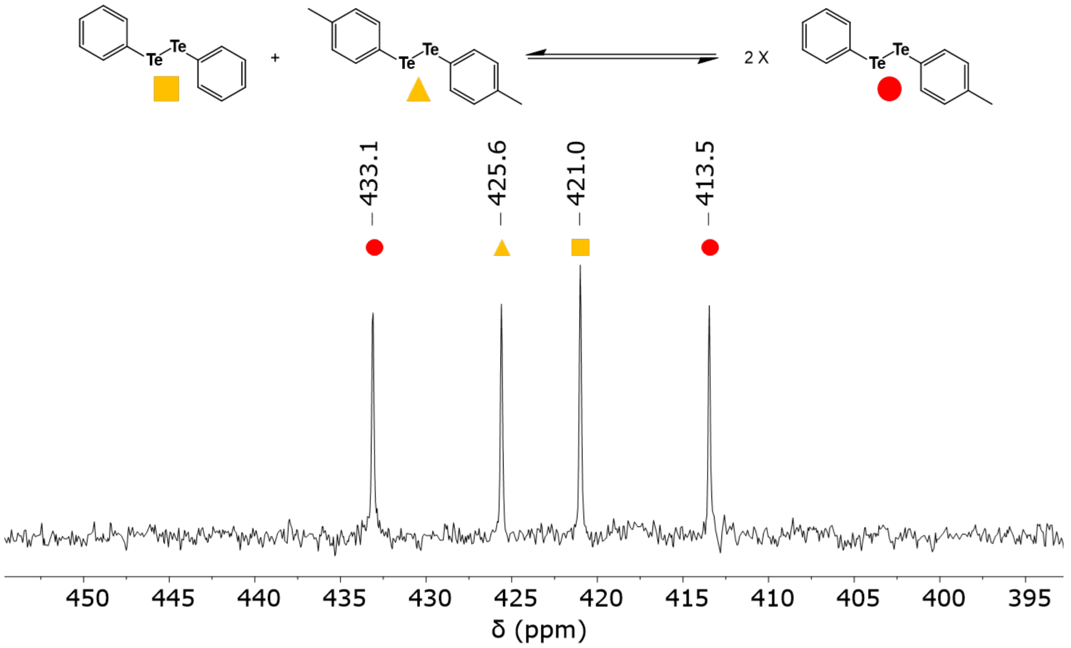

Figure S12. Dynamic Te-Te exchange of ditellurides. The ^{125}Te NMR spectrum showing the formation of the hetero-ditelluride from the corresponding homo-ditellurides by using Ph_2Te_2 and (*p*-MePh)$_2$$Te_2$ within 20 min at room temperature in acetone-d6.

S3.5 Diaryl ditelluride equilibrium at -50 °C

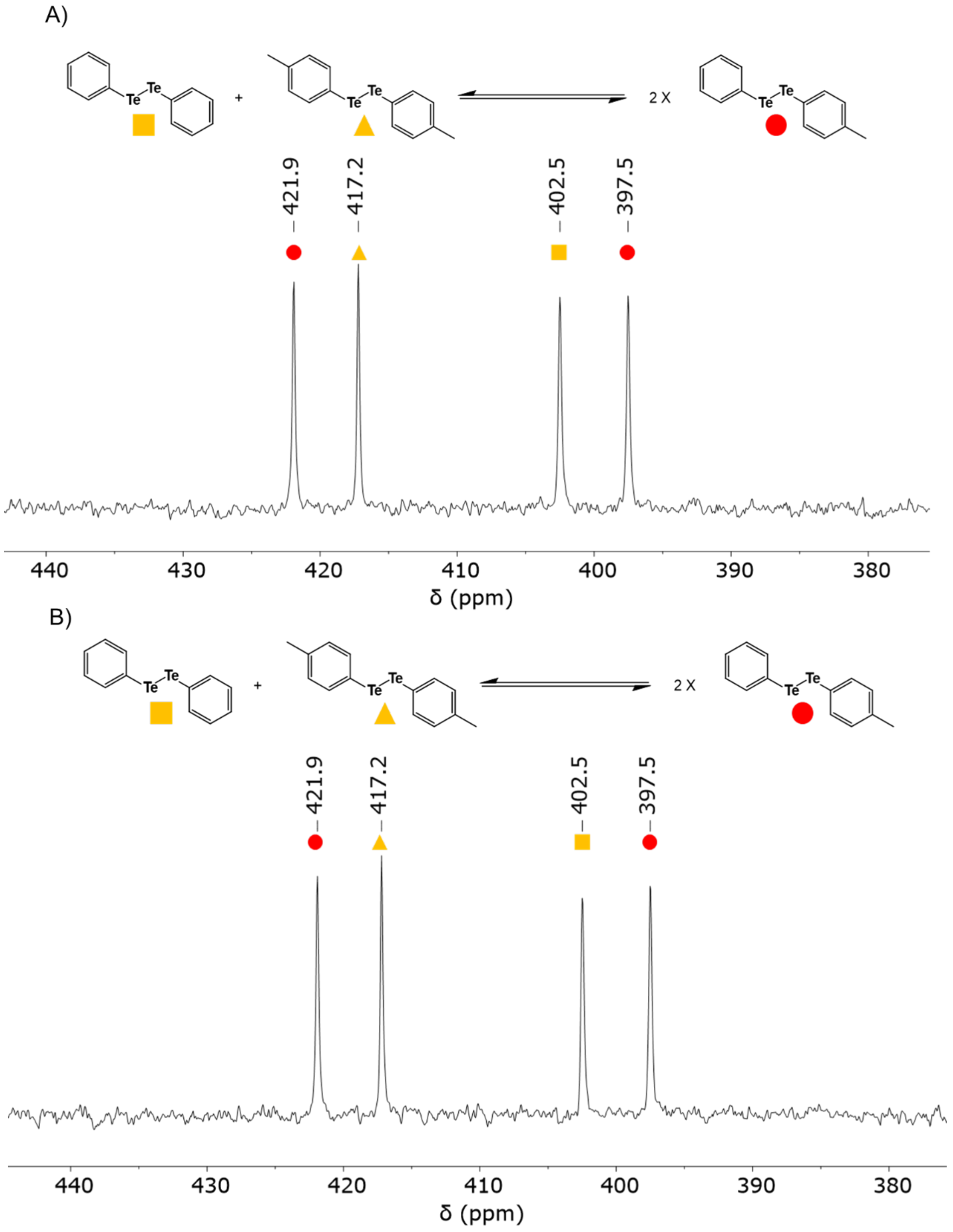

Figure S13. Dynamic Te-Te exchange of ditellurides. ^{125}Te NMR spectra showing the formation of the hetero-ditelluride from the corresponding homo-ditellurides by using Ph_2Te_2 and $(p\text{-}MePh)_2Te_2$ at -50 °C showing no significant difference in reactivity, but shows shift in signals A) after 20 min at -50 °C in $CDCl_3$, and B) after 1 h at -50 °C in $CDCl_3$.

S3.6 Diaryl ditelluride equilibrium in the absence of light

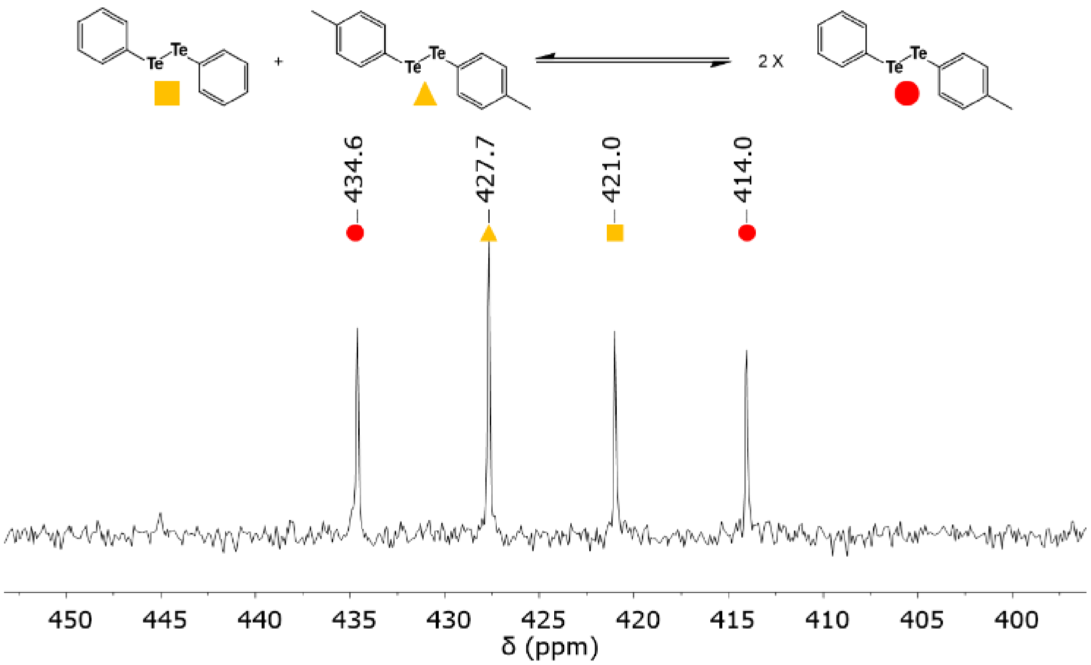

Figure S14. Dynamic Te-Te exchange of ditellurides. The ^{125}Te NMR spectrum showing the formation of the hetero-ditelluride from the corresponding homo-ditellurides by using Ph_2Te_2 and (*p*-MePh)$_2Te_2$ within 20 min at room temperature in $CDCl_3$ in the absence of visible light using a brown NMR tube.

S3.7 Diaryl ditelluride equilibrium with TEMPO radical scavenger

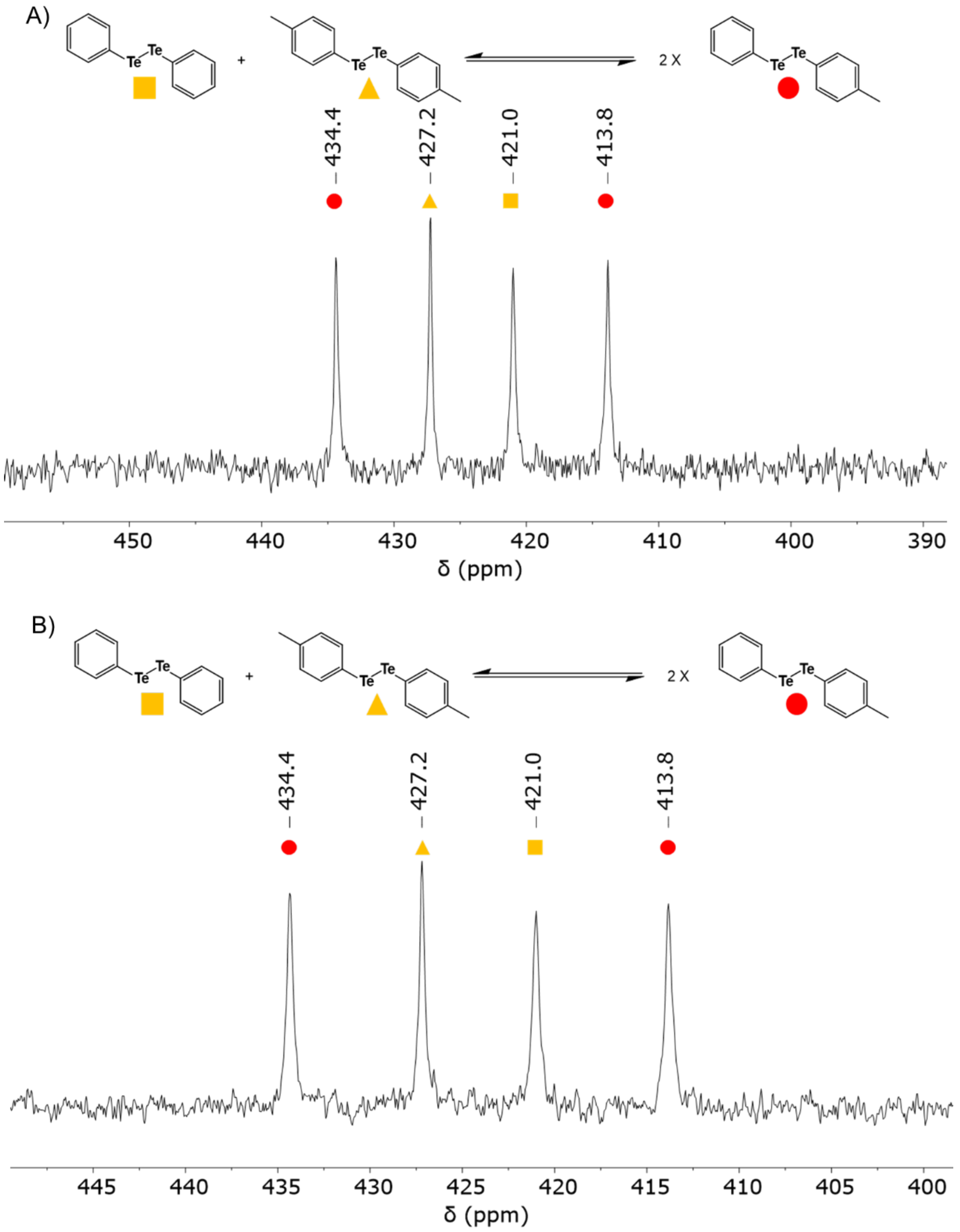

Figure S15. Dynamic Te-Te exchange of ditellurides. ^{125}Te NMR spectra showing the formation of the hetero-ditelluride from the corresponding homo-ditellurides by using Ph_2Te_2 and (p-MePh)$_2$Te$_2$ with the addition of 2 equivalent of the radical scavenger TEMPO, proving the reaction is not driven by radical exchange. A) Equilibrium within 20 min. B) Equilibrium after 24 h at room temperature.

S4 Ditelluride and diselenide Dynamic Combinatorial Libraries

S4.1 Time-course of equilibrium product formation of ditelluride with diselenides

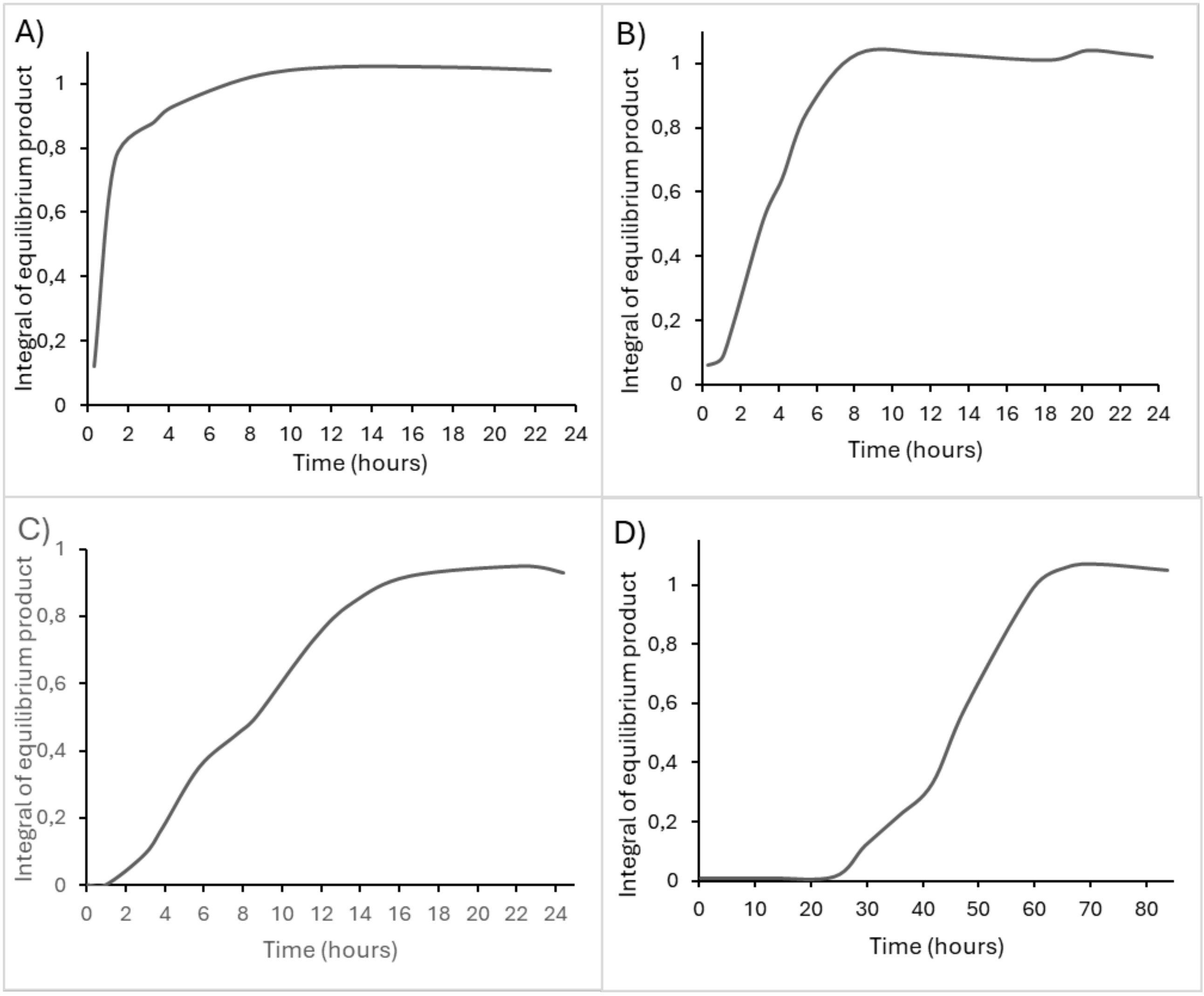

Figure S16. Line graphs depicting NMR integrals as a function of time of the Te-Te/Se-Se exchange reaction. The data illustrate changes in the integral values relative to the integral of Ph_2Te_2, indicating the formation of equilibrium product over time. A) ^{125}Te NMR spectrum of molar equivalent mixture of Ph_2Te_2 and Ph_2Se_2 over the course of 24 h forming equilibrium already after ~5 h. B) ^{125}Te NMR spectrum of molar equivalent mixture of Ph_2Te_2 and Ph_2Se_2 under the absence of visible light over the course of 24 h forming equilibrium after ~8 h. C) ^{125}Te NMR spectrum of molar equivalent mixture of Ph_2Te_2 and Me_2Se_2 over the course of 24 h forming equilibrium after ~18 h. C) ^{125}Te NMR spectrum of molar equivalent mixture of Me_2Te_2 and Me_2Se_2 over the course of 80 h forming equilibrium after ~65 h.

S4.2 Ditelluride and diselenide equilibrium formation with the absence of light

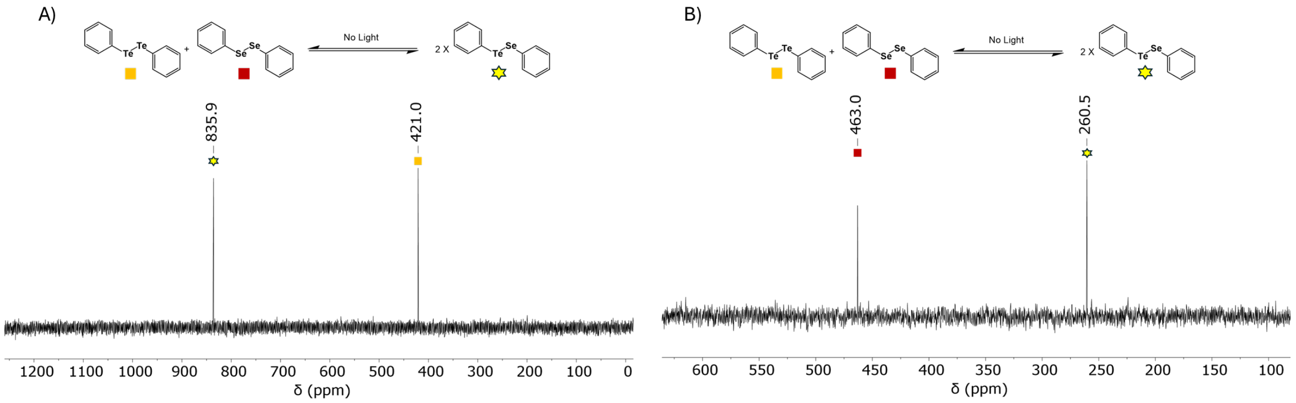

Figure S17. NMR data showing the full equilibration and exchange between a molar equivalent mixture of Ph_2Te_2 and Ph_2Se_2 after 24 hours at room temperature in $CDCl_3$ in the absence of visible light using a brown NMR tube. A) ^{125}Te NMR spectrum showing the formation of PhTeSePh. B) ^{77}Se NMR spectrum showing the formation of PhTeSePh.

S4.3 Addition of another ditelluride species to an equilibrium product formation of ditelluride with diselenides

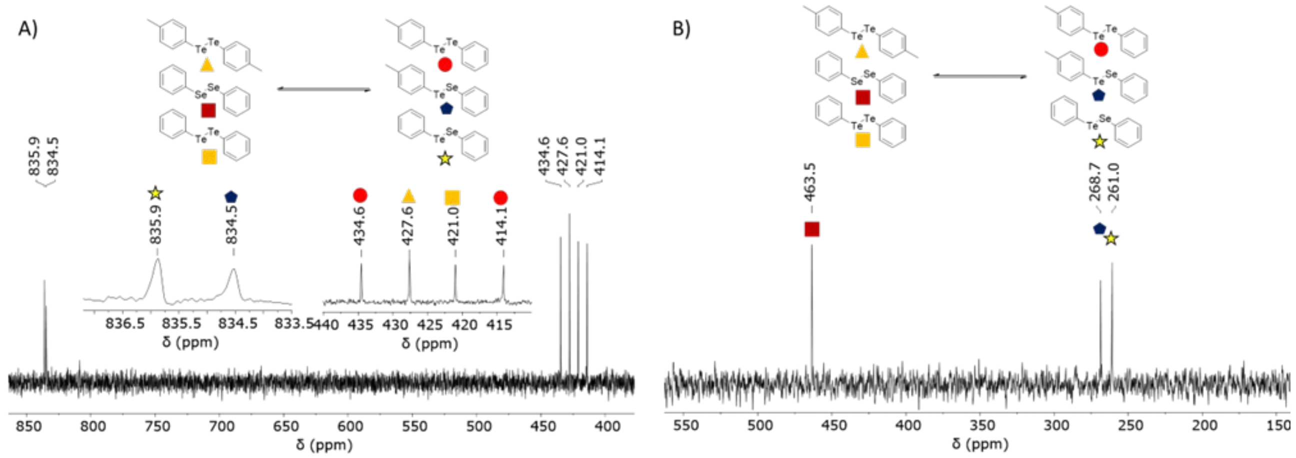

Figure S18. ^{125}Te NMR spectra showing the addition of an equimolar amount of (p-$MePh_2$)Te_2 to a preequilibrated mixture of Ph_2Te_2, Ph_2Se_2 and PhTeSePh, resulting in rapid configuration of equilibrium within 20 min at room temperature. A) The ^{125}Te NMR spectrum of the new equilibrium. B) The ^{77}Se NMR spectrum of the new equilibrium.

S5 Ditelluride and disulfide Dynamic Combinatorial Libraries

S5.1 Time-course of equilibrium product formation of ditelluride with disulfides

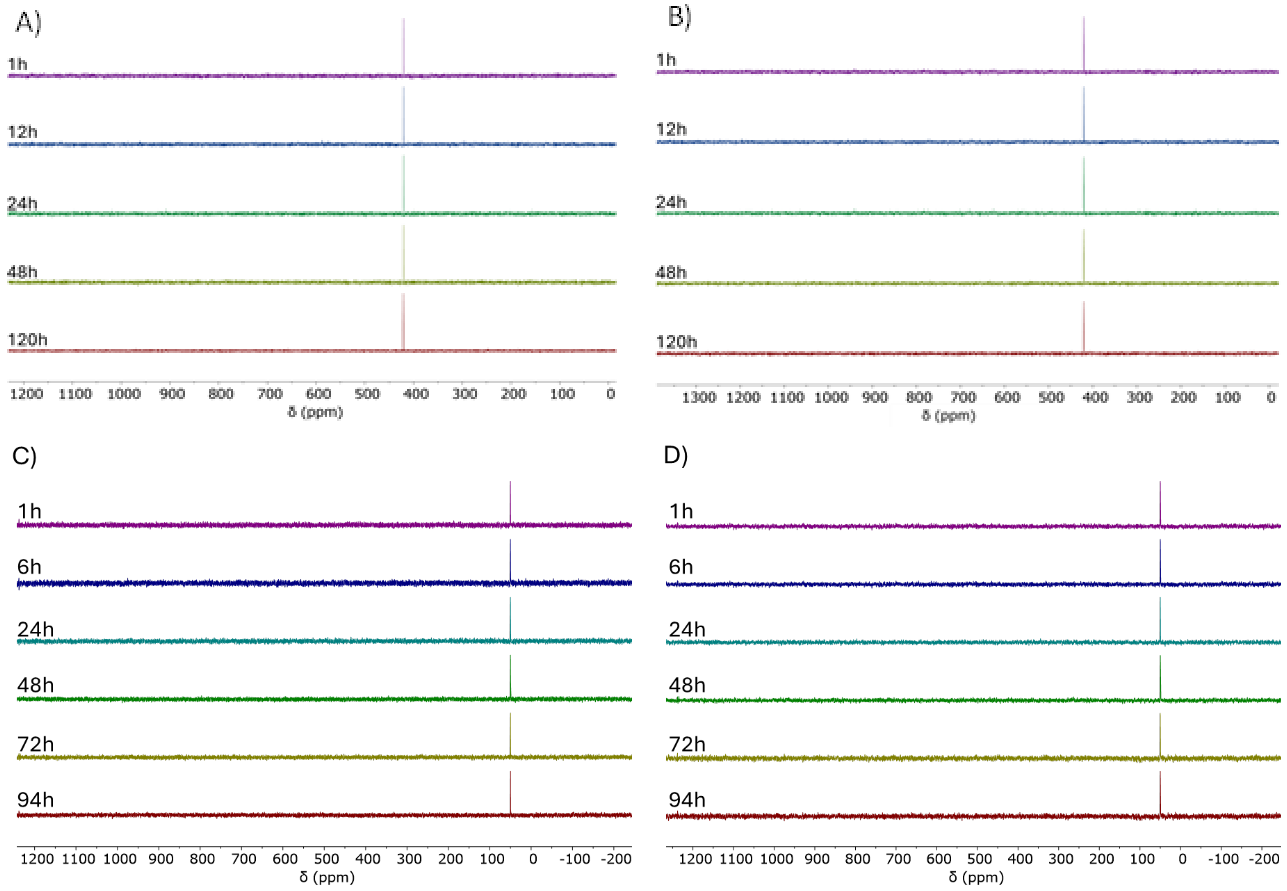

Figure S19. ^{125}Te NMR spectra time-course of the ditelluride-disulfide systems at room temperature in $CDCl_3$. A) ^{125}Te NMR spectra showing the time-course of molar equivalent mixture of Ph_2Te_2 and Ph_2S_2 from 1 to 120 h. B) ^{125}Te NMR spectra showing the time-course of molar equivalent mixture of Ph_2Te_2 and Ph_2S_2 in the presence of 20% molar equivalent of thiophenol from 1 to 120 h. C) ^{125}Te NMR spectra showing the time-course of molar equivalent mixture of Me_2Te_2 and Me_2S_2 from 1 to 92 h. D) ^{125}Te NMR spectra showing the time-course of molar equivalent mixture of Me_2Te_2 and Me_2S_2 in the presence of 20% molar equivalent of phenylthiol from 1 to 94 h.

S5.2 Attempted ditelluride and disulfide equilibrium formation

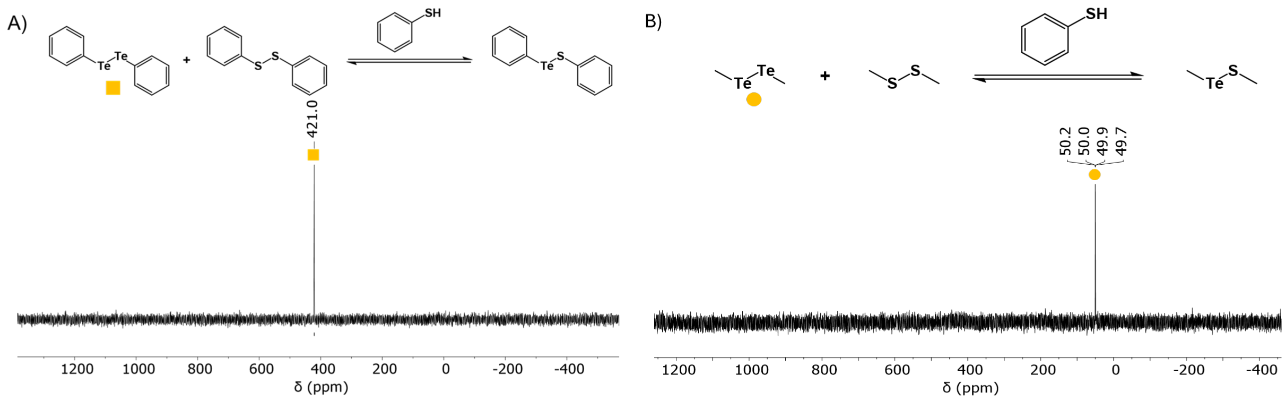

Figure S20. ^{125}Te NMR spectra of ditelluride-disulfide systems containing aromatic and aliphatic substituents, showing no equilibrium forming. A) ^{125}Te NMR spectrum of molar equivalent mixture of Ph_2Te_2 and Ph_2S_2 in the presence of 20% molar equivalent of phenylthiol after 120 h. B) ^{125}Te NMR spectrum of molar equivalent mixture of Me_2Te_2 and Me_2S_2 in the presence of 20% molar equivalent of phenylthiol after 94 h.

S6 Quantum chemical analyses

Table S2. Cartesian coordinates (in Å) and ADF total energy (in kcal mol^{-1}). Computed at ZORA-BLYP-D3(BJ)/TZP level of theory using COSMO to simulate solvation in chloroform.

Ph_2Te_2 (-3281.3)

1	C	-3.54507309	1.91319323	1.01661096
2	C	-4.26401421	1.32585365	-0.03425118
3	C	-3.58888263	0.67015503	-1.07306288
4	C	-2.18891055	0.60662074	-1.05718300
5	C	-1.46310733	1.19779279	-0.01316698
6	C	-2.14240793	1.84878559	1.02371201
7	H	-5.35225041	1.37638796	-0.03298318
8	H	-4.14901435	0.21082509	-1.88594811
9	H	-1.65444351	0.09883785	-1.85937754
10	H	-0.37618834	1.13564436	-0.00913515
11	Te	-1.11193263	2.85186649	2.66655199
12	H	-4.08406611	2.40999818	1.82257423
13	Te	1.39488098	1.69670464	2.40126302
14	C	2.23093404	3.01686196	0.87674218
15	C	3.62705674	3.01944188	0.72727049
16	C	4.21957553	3.81208187	-0.26621409
17	C	3.42493257	4.60625573	-1.10438192
18	C	2.03234294	4.60199430	-0.94627829
19	C	1.43233410	3.80549310	0.03943032
20	H	4.25812353	2.41678191	1.37939963
21	H	5.30348512	3.81281785	-0.37565925
22	H	3.88719888	5.22522043	-1.87166132
23	H	1.40577038	5.21613933	-1.59214072
24	H	0.35004018	3.81557952	0.15560498

(p-MePh)$_2$Te$_2$ (-4016.3)

1	C	-3.70404107	2.07133824	-1.43873993
2	C	-4.37721813	1.39402039	-0.41151283
3	C	-3.67482925	0.68820102	0.57947378
4	C	-2.26904923	0.68640503	0.51466966
5	C	-1.58516683	1.36572526	-0.50036725
6	C	-2.30272920	2.05871343	-1.48329346
7	H	-5.46717892	1.41179427	-0.38970531
8	H	-3.97628654	0.16016576	2.66064049
9	H	-1.69580898	0.14967643	1.27152571
10	H	-0.49690378	1.33908027	-0.52465751
11	Te	-1.33380570	3.20278341	-3.07161279
12	H	-4.28189710	2.59865889	-2.19716214
13	Te	1.16122246	1.99348956	-3.03115995
14	C	2.09795675	3.18450452	-1.45841738
15	C	3.50125276	3.19964902	-1.40578906
16	C	4.15477884	3.91028246	-0.39195261
17	C	3.43197554	4.62378672	0.58236838
18	C	2.02941549	4.60076043	0.50766633
19	C	1.36273895	3.88548618	-0.49759082
20	H	4.09226021	2.67015128	-2.15243231
21	H	5.24508076	3.91705159	-0.36723594
22	H	4.93106378	6.03446229	1.26371865
23	H	1.44276652	5.14792819	1.24613999
24	H	0.27447159	3.89598784	-0.53045267

25	C	4.15009186	5.38369227	1.67856573
26	H	3.45323247	6.00508754	2.25208034
27	H	4.64161122	4.69304767	2.37853927
28	C	-4.40147665	-0.06588403	1.67407141
29	H	-5.46725036	0.18825637	1.69132298
30	H	-4.31640809	-1.15190978	1.52538380

$PhTe_2$(*p*-MePh) (-3649.6)

1	C	-2.66117044	1.41731000	-0.77645832
2	C	-2.56506937	0.47747789	0.25873486
3	C	-1.81169967	0.76654682	1.40603181
4	C	-1.15535302	1.99885492	1.51844679
5	C	-1.24967393	2.94394594	0.48744671
6	C	-2.00230584	2.65206799	-0.66146213
7	H	-3.07702407	-0.47968877	0.16690246
8	H	-1.73643011	0.03317783	2.20746378
9	H	-0.56371365	2.22581021	2.40425986
10	H	-0.72768033	3.89361371	0.57189801
11	Te	-2.08323459	4.10999173	-2.26466276
12	H	-3.23903406	1.18572870	-1.66867531
13	Te	0.26944378	3.17128940	-3.52257989
14	C	1.56223004	3.67244902	-1.85962338
15	C	1.81427396	2.70852761	-0.87059568
16	C	2.61449545	3.02893048	0.23216987
17	C	3.17402248	4.31045511	0.37664519
18	C	2.91669007	5.26400626	-0.62552044
19	C	2.11975785	4.95401094	-1.73421760
20	H	1.37827960	1.71575815	-0.95081805
21	H	2.79863654	2.27177615	0.99456660
22	H	4.66114055	3.82741658	1.87828160
23	H	3.34576479	6.26251351	-0.53901275
24	H	1.93098899	5.71112533	-2.49253484
25	C	4.00720340	4.66012137	1.59143264
26	H	4.62855939	5.54389050	1.40755869
27	H	3.36198051	4.88017368	2.45447259

TS_$PhTe_2$(*p*-MePh) (-7289.3)

1	H	2.55066026	-2.81068683	1.81631190
2	H	-1.85947661	2.75659693	3.64103708
3	Te	0.35459852	-0.95273288	3.23066899
4	Te	-3.42595386	0.47966641	2.08571993
5	H	2.87156174	3.08687909	6.16101407
6	Te	-1.91390424	2.55637918	0.31349472
7	H	2.42308992	5.37742523	-1.04664280
8	H	2.53902614	1.45993402	4.33487890
9	H	0.42702203	4.81419644	0.30488500
10	Te	0.85292472	1.30603594	1.63526988
11	C	-0.07320988	3.04630536	-0.84972477
12	C	0.30344804	2.23676531	-1.93585370
13	C	1.43891428	2.55301096	-2.69384780
14	C	2.20603930	3.68332264	-2.37600056
15	C	1.83285653	4.49679442	-1.29811350
16	C	0.69984653	4.17779366	-0.53453193
17	H	-0.28506730	1.35565113	-2.18667417
18	H	1.72402352	1.91778515	-3.53203848
19	H	3.08925537	3.92731796	-2.96434538
20	C	1.15909907	2.72810011	3.24259683
21	C	0.50293793	3.96548537	3.21137898

22	C	0.71236251	4.88583376	4.24224694
23	C	1.56351790	4.59024142	5.32331765
24	C	2.21066158	3.34328395	5.33345965
25	C	2.02280814	2.41673363	4.29977026
26	H	-0.19537069	4.20059808	2.41168771
27	H	0.18977684	5.84196007	4.21246762
28	H	2.45714422	5.20743509	7.19843538
29	C	-2.72859426	0.83276622	4.10184563
30	C	-2.91415943	-0.14329978	5.09805790
31	C	-2.37410897	0.04681369	6.37579427
32	C	-1.62594750	1.19885405	6.66585553
33	C	-1.44381938	2.17116056	5.67475996
34	C	-2.00573933	1.99650658	4.40277056
35	H	-3.45895505	-1.05885673	4.87384445
36	H	-2.52360286	-0.71474149	7.14085572
37	H	-1.18813523	1.33286127	7.65391956
38	H	-0.85769902	3.06456431	5.88045406
39	C	0.52806933	-2.13539833	1.42305279
40	C	-0.54760577	-2.16367003	0.52481679
41	C	-0.43527783	-2.88962915	-0.66817768
42	C	0.73395575	-3.60375211	-0.97936490
43	C	1.80034747	-3.56701857	-0.06065483
44	C	1.70730351	-2.83792887	1.12989781
45	H	-1.46210227	-1.61662020	0.74916321
46	H	-1.27235724	-2.89743278	-1.36621961
47	H	1.79238140	-4.19040944	-2.77636108
48	H	2.72044382	-4.10835034	-0.28212509
49	C	0.84718917	-4.40253563	-2.26025566
50	H	0.82787111	-5.48124299	-2.04949119
51	H	0.02080363	-4.17793351	-2.94344026
52	C	1.75801845	5.58695331	6.44557122
53	H	2.14810989	6.53955923	6.06265512
54	H	0.80381461	5.80628360	6.94406131

Ph_2Se_2 (-3314.5)

1	C	-3.15338889	1.94073465	0.95854327
2	C	-3.87490498	1.29603195	-0.05494615
3	C	-3.20445563	0.56618288	-1.04683933
4	C	-1.80562659	0.48435274	-1.02003177
5	C	-1.07390296	1.13104573	-0.01422279
6	C	-1.75251814	1.85367409	0.97219918
7	H	-4.96204962	1.36234445	-0.06259546
8	H	-3.76776041	0.06417522	-1.83158220
9	H	-1.27566699	-0.08063738	-1.78581420
10	H	0.01145359	1.06127146	0.00446802
11	Se	-0.83632572	2.85067191	2.40784123
12	H	-3.68315433	2.49902237	1.72970020
13	Se	1.36584366	1.97734516	2.32883719
14	C	2.21674150	3.06440066	0.91837764
15	C	3.61381981	2.96674564	0.82410328
16	C	4.28890488	3.67614446	-0.17803272
17	C	3.57630118	4.48120785	-1.07817770
18	C	2.18185773	4.57222889	-0.97156940
19	C	1.49611277	3.86116552	0.02313335
20	H	4.17653816	2.35014202	1.52428476
21	H	5.37327546	3.60138205	-0.24822305
22	H	4.10376480	5.03361712	-1.85390648
23	H	1.61929314	5.19507310	-1.66592226

24	H	0.41410716	3.93837871	0.10459300

PhTeSePh (-3298.6)

1	C	-3.30810453	1.87856615	1.02561551
2	C	-3.99040840	1.18394880	0.01705643
3	C	-3.28295520	0.60398478	-1.04466362
4	C	-1.88727752	0.72714523	-1.09746552
5	C	-1.19783336	1.42968272	-0.10033315
6	C	-1.91134711	1.99929306	0.96140675
7	H	-5.07396120	1.08770329	0.07227583
8	H	-3.81395965	0.05775841	-1.82243041
9	H	-1.32902045	0.28030925	-1.91916927
10	H	-0.11515258	1.51914031	-0.14991969
11	Se	-1.05483033	3.07497185	2.37661757
12	H	-3.86263516	2.31227696	1.85654392
13	Te	1.29181673	2.04437604	2.54606035
14	C	2.26260789	3.16464297	0.94200206
15	C	3.63711447	2.95263980	0.74973552
16	C	4.30727318	3.63022061	-0.27834757
17	C	3.61115026	4.51589402	-1.11316387
18	C	2.23959153	4.72206902	-0.91310251
19	C	1.56204395	4.04779529	0.11315256
20	H	4.19217224	2.26820286	1.39085180
21	H	5.37413239	3.46369307	-0.42334095
22	H	4.13368284	5.04053165	-1.91147071
23	H	1.68931752	5.40813601	-1.55623200
24	H	0.49716943	4.21675752	0.26576923

TS_PhTeSePh (-6585.7)

1	H	2.06852680	-3.59926164	2.43960803
2	H	-1.28947683	1.51565703	3.58205788
3	Se	0.50348613	-1.08610627	2.85144687
4	Se	-2.18267780	-1.31293675	2.94948812
5	H	2.44022271	2.28710315	6.52797652
6	Te	-1.55292788	1.60356503	0.58406938
7	H	-0.35548557	6.64734709	-0.37364482
8	H	2.47548258	0.80192885	4.54165077
9	H	-1.53181881	4.76757797	0.73588189
10	Te	1.54421649	1.00344563	1.48852577
11	C	-0.54108808	3.23107017	-0.42611035
12	C	0.40812852	2.98289741	-1.43614308
13	C	1.08166500	4.04648412	-2.05282140
14	C	0.81023637	5.36884156	-1.67519754
15	C	-0.13821864	5.62275000	-0.67393039
16	C	-0.80588364	4.56096470	-0.04853867
17	H	0.61884415	1.96113558	-1.74528764
18	H	1.81470441	3.84002022	-2.83218718
19	H	1.33285837	6.19410776	-2.15652917
20	C	1.51552775	2.25595534	3.24994794
21	C	0.96338609	3.54128320	3.14781249
22	C	0.95765134	4.37806342	4.27087778
23	C	1.49129014	3.93035918	5.48728995
24	C	2.03346930	2.64245115	5.58271483
25	C	2.05231480	1.80046916	4.46297196
26	H	0.53583754	3.88623977	2.21007579
27	H	0.53064335	5.37679704	4.19421058
28	H	1.47856567	4.58145964	6.35961527
29	C	-2.18434551	-0.20163853	4.55176923

30	C	-2.68420592	-0.72228911	5.75931734
31	C	-2.67161424	0.06631817	6.91677731
32	C	-2.14272293	1.36564644	6.88330925
33	C	-1.63622598	1.87767117	5.68163677
34	C	-1.66097256	1.10276460	4.51583191
35	H	-3.07720238	-1.73645152	5.79361842
36	H	-3.06514755	-0.34058510	7.84755789
37	H	-2.12385148	1.97099781	7.78829381
38	H	-1.21441978	2.87966300	5.64479058
39	C	0.70486627	-2.32644289	1.34151865
40	C	0.01072661	-2.10774526	0.14388649
41	C	0.16176164	-3.02087557	-0.90715521
42	C	0.99202019	-4.14086137	-0.75822821
43	C	1.67625228	-4.35098341	0.44626053
44	C	1.53589780	-3.44231200	1.50456481
45	H	-0.63362295	-1.23931038	0.02629687
46	H	-0.37319632	-2.85525629	-1.84097127
47	H	1.10525864	-4.84735709	-1.57876144
48	H	2.32236626	-5.21905485	0.56620985

Ph_2S_2 (-3346.0)

1	C	-2.88447251	1.99698987	0.92698916
2	C	-3.63255576	1.33961798	-0.05632692
3	C	-2.99336658	0.55831417	-1.03002686
4	C	-1.59753828	0.43940305	-1.01356538
5	C	-0.83730370	1.09779792	-0.03778275
6	C	-1.48333444	1.87462371	0.93231008
7	H	-4.71736630	1.43572752	-0.05521669
8	H	-3.57853982	0.04598477	-1.79165347
9	H	-1.09071754	-0.16488719	-1.76464930
10	H	0.24525918	1.00078267	-0.02903465
11	S	-0.61910496	2.81304528	2.21913409
12	H	-3.38630289	2.59645484	1.68553895
13	S	1.33689458	2.10386604	2.21557317
14	C	2.19818357	3.04842968	0.93150844
15	C	3.59966721	2.92995671	0.92572588
16	C	4.34605948	3.59198460	-0.05577622
17	C	3.70479435	4.37435960	-1.02718314
18	C	2.30861364	4.48955075	-1.01028739
19	C	1.55014704	3.82654361	-0.03636137
20	H	4.10319627	2.32980424	1.68264359
21	H	5.43111778	3.49872465	-0.05493025
22	H	4.28853184	4.89030419	-1.78747264
23	H	1.80023869	5.09453601	-1.75975892
24	H	0.46737807	3.92077550	-0.02745402

PhTeSPh (-3313.3)

1	C	-3.31083532	2.04672056	1.02462950
2	C	-4.12024021	1.58118803	-0.02098423
3	C	-3.56538983	0.82244344	-1.06131256
4	C	-2.19527614	0.52850266	-1.04934600
5	C	-1.37911063	0.98795863	-0.00542623
6	C	-1.93942205	1.74711552	1.02791903
7	H	-5.18413927	1.81568047	-0.02014560
8	H	-4.19557070	0.46460820	-1.87390278
9	H	-1.75437484	-0.05999056	-1.85325538
10	H	-0.31599983	0.75295863	-0.00028900
11	Te	-0.74509031	2.52050135	2.68233062

12	H	-3.75607736	2.63888797	1.82382724
13	S	1.43442924	1.66414818	1.96241796
14	C	2.09553805	2.89490208	0.81397890
15	C	3.48843432	3.08830467	0.82964978
16	C	4.08069318	3.96741935	-0.08628071
17	C	3.29046300	4.66711050	-1.00774777
18	C	1.90081662	4.47742575	-1.01339873
19	C	1.30163230	3.58973264	-0.11270672
20	H	4.10255963	2.56045290	1.55720492
21	H	5.15971518	4.11399067	-0.06588677
22	H	3.75150422	5.35817660	-1.71133286
23	H	1.27803267	5.01729977	-1.72533894
24	H	0.22409199	3.44579542	-0.12688746

TS_PhTeSPh (-6611.4)

1	H	2.28035895	-2.94263044	2.29725416
2	H	-1.31573493	1.92542437	3.36955430
3	S	0.51135018	-0.67457468	2.54904549
4	S	-2.20801499	-0.79527826	2.78088524
5	H	2.51970163	2.37093434	6.38549125
6	Te	-1.29056017	2.01832506	0.35691578
7	H	0.20312664	6.91887276	-0.88479897
8	H	2.46044062	0.93759401	4.36042304
9	H	-1.01111762	5.17433397	0.39383296
10	Te	1.56888596	1.28300400	1.31788901
11	C	-0.24586810	3.53099379	-0.79301794
12	C	0.58082481	3.17293285	-1.87353964
13	C	1.27457727	4.16001765	-2.58684293
14	C	1.14131020	5.51047577	-2.23486520
15	C	0.31229947	5.87145042	-1.16365575
16	C	-0.37520683	4.88647528	-0.44118645
17	H	0.68357424	2.12796668	-2.15925613
18	H	1.91601840	3.87245829	-3.41915886
19	H	1.67966247	6.27595370	-2.79149168
20	C	1.59532112	2.48734768	3.11368223
21	C	1.12055909	3.80371540	3.04300911
22	C	1.16432241	4.60804269	4.18861357
23	C	1.67158806	4.09524542	5.39036248
24	C	2.13604113	2.77542411	5.45054748
25	C	2.10451697	1.96340407	4.30922649
26	H	0.71316040	4.19777314	2.11538191
27	H	0.79605118	5.63146439	4.14079738
28	H	1.07941262	-4.64321272	-1.48464609
29	C	-2.12083389	0.13889043	4.28939869
30	C	-2.51776516	-0.44320412	5.51643819
31	C	-2.43070840	0.28142281	6.70928747
32	C	-1.92849789	1.59272376	6.71000341
33	C	-1.52238644	2.17432206	5.50101555
34	C	-1.62159618	1.46111956	4.30288361
35	H	-2.89357397	-1.46471909	5.52280253
36	H	-2.74665692	-0.18270945	7.64326855
37	H	-1.85026236	2.14972923	7.64246114
38	H	-1.11870219	3.18457027	5.48744595
39	C	0.69909484	-1.90565223	1.24965108
40	C	-0.12302165	-1.87277160	0.10927803
41	C	0.02304039	-2.85708964	-0.87363640
42	C	0.97363605	-3.87676307	-0.71870940
43	C	1.78448365	-3.91114542	0.42373904

44	C	1.65244746	-2.92561933	1.40964136
45	H	-0.86197364	-1.08476376	-0.00630494
46	H	-0.61243522	-2.83121728	-1.75730792
47	H	1.69702055	4.72149703	6.28033809
48	H	2.52239397	-4.70185597	0.54881679

Table S3. Bonding, strain and interaction energies (in kcal mol^{-1}) of the chalcogen-chalcogen homolytic breaking. Chalcogen-chalcogen bond length is also included (in Å). Computed at ZORA-BLYP-D3(BJ)/TZ2P in $CHCl_3$.

System	Interaction	ΔE	ΔE_{int}	ΔE_{strain}	r
Ph_2Te_2	Te-Te	-44.1	-45.4	1.3	2.773
PhTeSePh	Te-Se	-45.7	-47.7	2.0	2.569
Ph_2Se_2	Se-Se	-45.8	-48.7	2.9	2.370
PhTeSPh	Te-S	-43.5	-46.1	2.5	2.450
Ph_2S_2	S-S	-43.7	-48.3	4.6	2.081

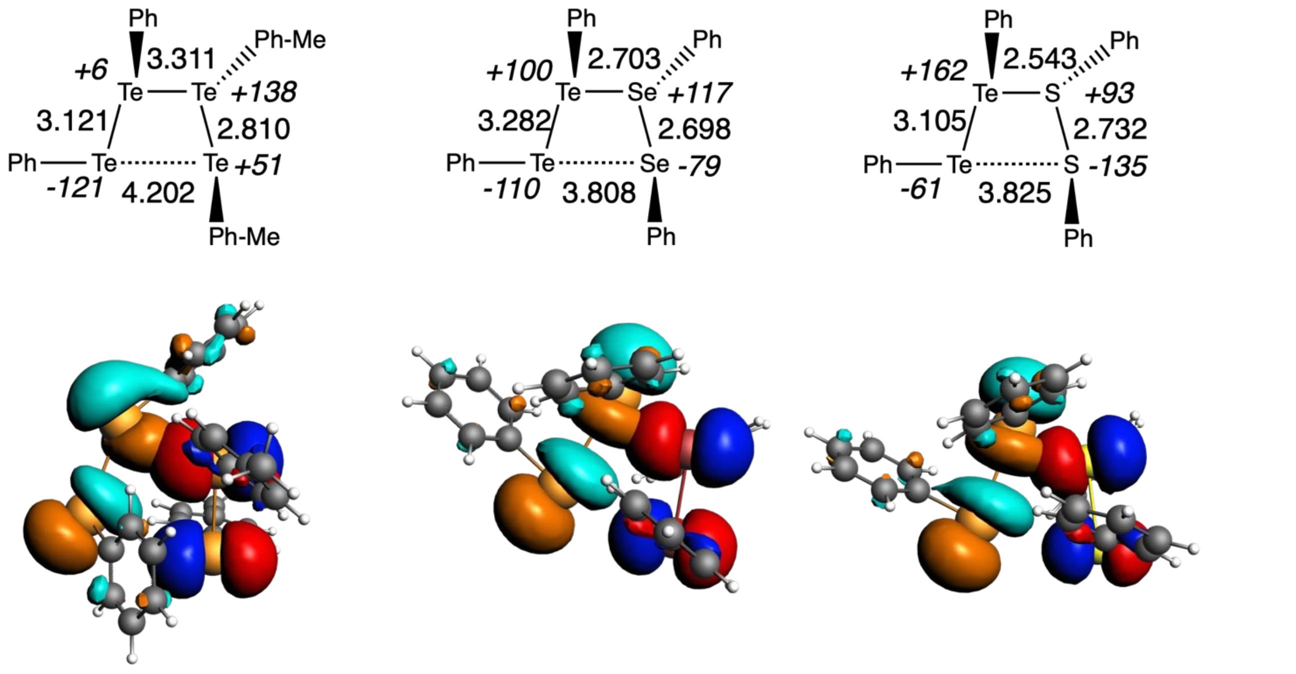

2 PhTeTe(*p*-MePh) 2 PhTeSePh 2 PhTeSPh

Figure S21. Bond lengths (in Å) and VDD charges (in milli-au, in italics) of the three transition states under analysis (top). And isosurfaces of the superposed HOMO and LUMO of the two fragments to build the TSs (isovalue = 0.03, bottom).

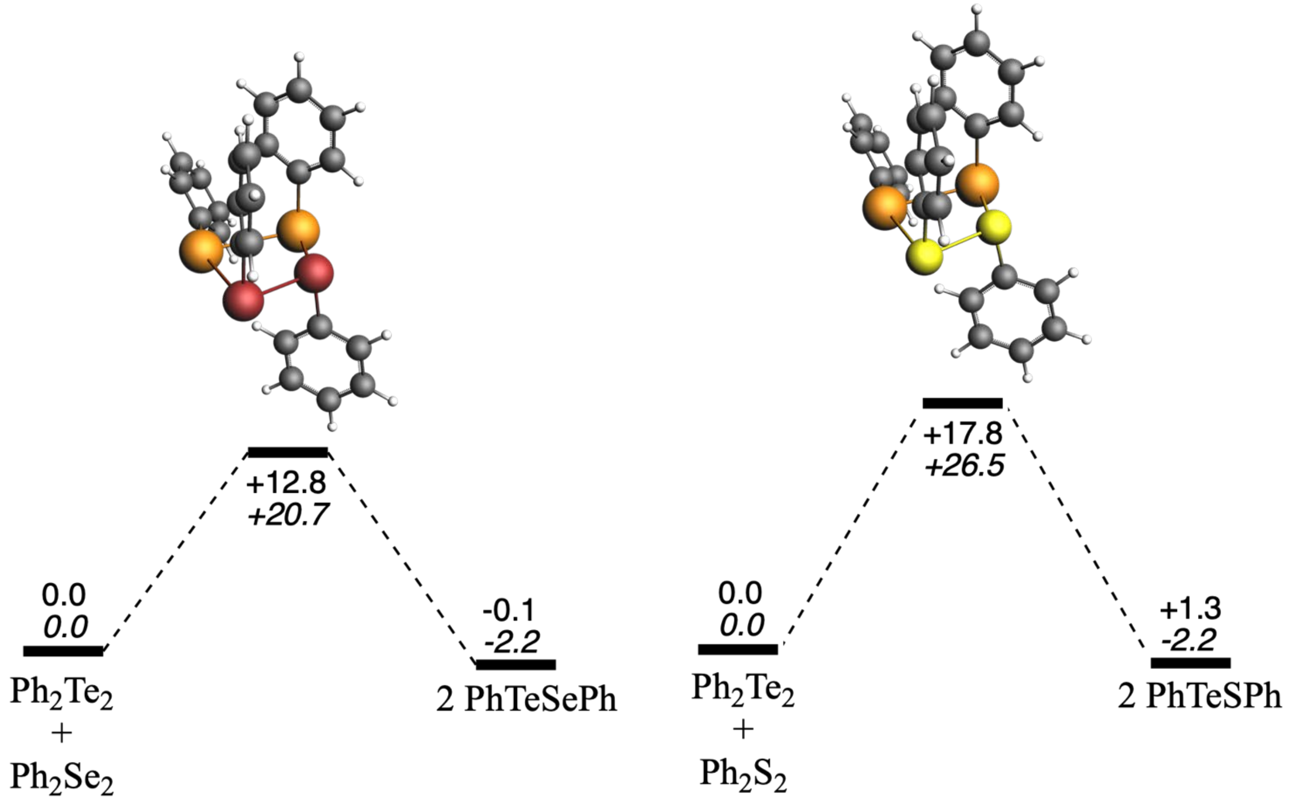

Figure S22. Proposed concerted mechanism for the equilibrium between diaryl ditelluride and diaryl diselenide (left) and diaryl disulfide (right). Enthalpies and Gibbs (in italics) energies are enclosed (in kcal mol^{-1}). Computed at ZORA-BLYP-D3(BJ)/TZP level of theory in chloroform.

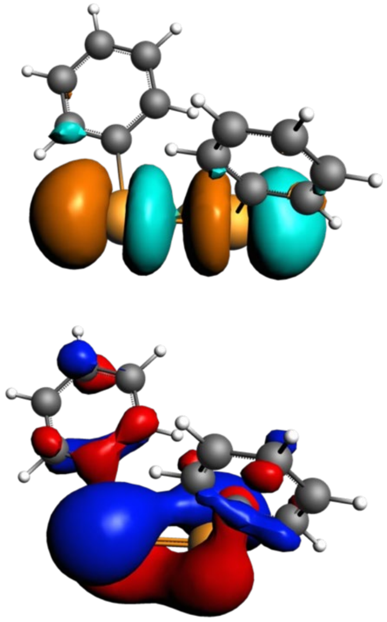

Figure S23. HOMO (lower) and LUMO (upper) orbitals (isovalue = 0.03) of Ph_2Te_2. Computed at ZORA-BLYP-D3(BJ)/TZP level of theory in chloroform.
